# The Role of Biomarkers in HPV-Positive Head and Neck Squamous Cell Carcinoma: Towards Precision Medicine

**DOI:** 10.3390/diagnostics14131448

**Published:** 2024-07-07

**Authors:** Antea Krsek, Lara Baticic, Vlatka Sotosek, Tamara Braut

**Affiliations:** 1 Faculty of Medicine, University of Rijeka, 51000 Rijeka, Croatia; tea.krsek@gmail.com; 2 Department of Medical Chemistry, Biochemistry and Clinical Chemistry, Faculty of Medicine, University of Rijeka, 51000 Rijeka, Croatia; 3 Department of Clinical Medical Sciences I, Faculty of Health Studies, University of Rijeka, 51000 Rijeka, Croatia; vlatkast@uniri.hr; 4 Department of Anesthesiology, Reanimatology, Emergency and Intensive Care Medicine, Faculty of Medicine, University of Rijeka, 51000 Rijeka, Croatia; 5 Department of Otorhinolaryngology and Head and Neck Surgery, Clinical Hospital Centre Rijeka, 51000 Rijeka, Croatia; tamara.braut@uniri.hr

**Keywords:** biomarkers, head and neck cancer, HPV diagnosis, HPV infection, squamous cell carcinoma

## Abstract

Head and neck cancer (HNC) represents a significant global health challenge, with squamous cell carcinomas (SCCs) accounting for approximately 90% of all HNC cases. These malignancies, collectively referred to as head and neck squamous cell carcinoma (HNSCC), originate from the mucosal epithelium lining the larynx, pharynx, and oral cavity. The primary risk factors associated with HNSCC in economically disadvantaged nations have been chronic alcohol consumption and tobacco use. However, in more affluent countries, the landscape of HNSCC has shifted with the identification of human papillomavirus (HPV) infection, particularly HPV-16, as a major risk factor, especially among nonsmokers. Understanding the evolving risk factors and the distinct biological behaviors of HPV-positive and HPV-negative HNSCC is critical for developing targeted treatment strategies and improving patient outcomes in this complex and diverse group of cancers. Accurate diagnosis of HPV-positive HNSCC is essential for developing a comprehensive model that integrates the molecular characteristics, immune microenvironment, and clinical outcomes. The aim of this comprehensive review was to summarize the current knowledge and advances in the identification of DNA, RNA, and protein biomarkers in bodily fluids and tissues that have introduced new possibilities for minimally or non-invasive cancer diagnosis, monitoring, and assessment of therapeutic responses.

## 1. Introduction

Roughly 90% of head and neck cancer (HNC) cases are squamous cell carcinomas. Head and neck squamous cell carcinoma (HNSCC) is a broad category of tumors that arise from the mucosal epithelium of the larynx, pharynx, and mouth cavity [1,2,3,4]. Although alcohol and tobacco have long been associated with HNSCC in impoverished nations, human papillomavirus (HPV) infection, particularly HPV-16, has been identified as a major risk factor in affluent nations, especially for nonsmokers [5]. As a result, HNSCC can be classified as HPV-positive or HPV-negative, with each showing unique molecular characteristics, immunological microenvironment, and clinical outcomes [6,7]. HPV-positive oropharyngeal cancer exhibits distinctive genetic profiles and a better prognosis in comparison to HPV-negative tumors, underscoring the significance of HPV status for prognosis in HNSCC [8]. Due to increased understanding of HPV’s role in the pathogenesis of oropharyngeal squamous cell carcinoma (OPSCC), two separate disease entities have been identified: HPV-positive disease and HPV-negative illness associated with risk factors such as alcohol and tobacco use. In addition to HPV, Epstein–Barr virus (EBV) is linked as well to a number of head and neck malignancies, including nasopharyngeal carcinoma [9].

HPV is an important etiological factor in the development of HNSCC, particularly oropharyngeal cancers [8,9]. This association is supported by intricate biological mechanisms that facilitate viral infection, integration, oncogene expression, immune evasion, cellular transformation, and tumor progression [1,5,9]. HPV is primarily transmitted through direct contact, mostly sexual contact, including oral sex. The virus infects the basal epithelial cells of the mucosal surfaces in the oropharynx, gaining entry through micro-abrasions or micro-wounds in the epithelial lining [4,6]. HPV can persist in the host cells for a long time, with its genome sometimes integrating into the host cell DNA. This integration is a pivotal event that disrupts the normal regulatory mechanisms of the host cell, particularly affecting the viral E2 gene. The disruption of E2 leads to the unregulated expression of the oncogenes E6 and E7, which are critical to HPV’s oncogenic potential [1,2,7]. E6 promotes the degradation of the tumor suppressor protein p53 through the ubiquitin–proteasome pathway. Under normal conditions, p53 plays a crucial role in inducing cell cycle arrest and apoptosis in response to DNA damage. The degradation of p53 by E6 results in uncontrolled cell division and reduced apoptosis. Additionally, E6 activates telomerase reverse transcriptase (TERT), extending the telomeres of cells and contributing to cellular immortality. Simultaneously, the E7 oncoprotein binds to and inactivates the retinoblastoma (Rb) protein [4,7,8]. Rb typically controls cell cycle progression at the G1–S checkpoint, and its inactivation leads to unregulated cell cycle progression and proliferation. E7 also interacts with and activates cyclin-dependent kinases, further promoting cell cycle progression. The interplay of these viral oncoproteins disrupts normal cell cycle control and DNA-repair mechanisms, leading to genomic instability and an increased likelihood of additional mutations [7]. HPV infection can induce changes in epithelial cells that promote epithelial-to-mesenchymal transition (EMT), characterized by increased cell motility and invasiveness, which are critical steps in cancer metastasis. The virus also alters the expression of genes involved in cell proliferation, apoptosis, and DNA repair, further contributing to oncogenesis [6,7,8].

HNSCC is a multifaceted disease influenced by various risk factors, HPV emerging as a significant etiological agent, particularly for oropharyngeal cancers. Studies show that patients with HPV-positive HNSCC frequently have higher immune cell infiltration, especially T cells, associated with a lower neo-antigen mutational burden [10]. Because of this feature, HPV-positive HNSCCs are considered “hot tumors” and are amenable to therapies such as immunotherapy, chemotherapy, and radiation therapy [11,12].

More than 660,000 new cases of head and neck cancers are identified annually, making it the seventh-most common malignancy worldwide [3]. Because smoking rates are dropping and HPV-positive tumors are becoming more common, particularly in the oropharynx, the epidemiology of HNC is changing globally [13,14,15]. HPV has significantly altered the landscape of HNSCC epidemiology, introducing distinct risk factors and mechanisms of carcinogenesis. Understanding these factors is crucial for developing targeted prevention, screening, and treatment strategies to combat the rising incidence of HPV-associated HNSCC.

**Figure 1:**
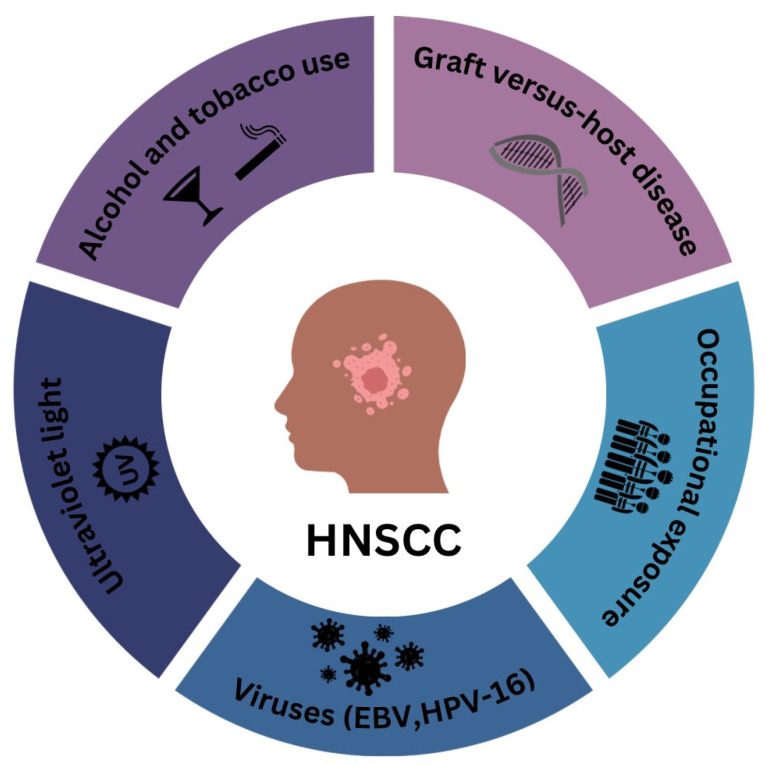
Risk factors for the development of head and neck squamous cell carcinoma. The image outlines the risk factors for head and neck squamous cell carcinoma (HNSCC), including alcohol and tobacco use, graft-versus-host disease, ultraviolet (UV) light, viruses, and occupational exposure. Alcohol and tobacco use are major risk factors, as they contain carcinogens that can lead to cellular mutations and cancer. Graft-versus-host disease creates a proinflammatory environment where donor immune cells attack the recipient’s tissues after an allogeneic stem cell transplant. This chronic inflammation and immune dysregulation increase the risk of secondary malignancies like HNSCC. Ultraviolet (UV) light causes direct DNA damage, leading to mutations and cancer. Viruses such as Epstein–Barr Virus (EBV) and human papillomavirus (HPV-16) are known to induce oncogenic transformations in cells, significantly raising the risk of HNSCC. Occupational exposure involves contact with carcinogenic substances in certain industries, which raises the risk of cancer development. Examples include asbestos fibers, which increase HNSCC risk, wood dust exposure in carpentry, which raises the risk of nasopharyngeal cancer, and exposure to industrial chemicals like formaldehyde and solvents in the textile, rubber, and plastic industries, as well as nickel refining and processing.

Historically, HNSCC has been associated with several well-established risk factors. Tobacco use is considered the leading cause, with the carcinogens in tobacco smoke causing DNA damage that promotes cancer development. This risk is further amplified when combined with heavy alcohol consumption, as the toxic metabolite acetaldehyde exacerbates the harmful effects. Poor oral hygiene, often leading to chronic inflammation and infections, also contributes to the risk. Nutritional deficiencies, particularly diets low in fruits and vegetables, are another contributing factor due to the lack of protective antioxidants. Additionally, occupational exposures to certain chemicals and dusts, such as asbestos and wood dust, have been linked to an increased risk of HNSCC [1,2,3,4]. However, the emergence of HPV, particularly HPV type 16, as a significant risk factor for oropharyngeal cancers, has shifted the epidemiological profile of HNSCC. The primary route of HPV transmission to the oropharyngeal region is through oral sex, making sexual behavior a critical factor. Individuals with multiple sexual partners or those who engage in sexual activity at a younger age are at higher risk of HPV infection. Furthermore, the sexual history of one’s partner can also influence risk, as partners with multiple previous sexual partners may increase the likelihood of HPV transmission [1,2,3,4,8,12]. The most common risk factors for the development of HNSCC are presented in Figure 1.

Given the crucial aspects of HPV in the etiopathogenesis of several important cancers and challenges in HPV diagnosis, the aim of this review was to compile information about the methods implemented to detect, at the level of DNA, RNA and proteins, HPV prevalence, and viral load, as well as molecules altered by HPV that have shown advantages in the search for biomarkers for diagnosis, prognosis, monitoring, and response to treatment in HNSCC. Studies are discussed with results of sensitivity and specificity of methods such as circulating tumor DNA as liquid biopsy in plasma and saliva, circulating tumor tissue-modified viral (cTTMV) HPV DNA through digital droplet PCR (ddPCR), HPV E6, and E7 mRNA. The use of miRNA profiles and some lncRNAs associated with HPV positivity as prognostic indicators or predictive biomarkers are also discussed, as well as the correlation of the p16INK4a indicator with fine-needle aspiration-tested E6 oncoprotein, E6/E7 mRNA, and HPV DNA. Moreover, new technologies in HPV-positive OPSCC, such as CRISPR-based diagnostics, next-generation sequencing and nanodiagnostics, are described, aiming to detect HPV viral load and to identify molecular characteristics associated with HPV positivity impacting diagnosis, prognosis, or recurrence, including copy-number variations, mutations driven by HPV integration, and immune cell tumor infiltration.

## 2. Molecular Biomarkers in HPV-Positive Head and Neck Cancer: A Focus on DNA, RNA, and Protein Indicators

Bodily fluids have become an important material for cancer detection across many anatomical locations because of the non-invasive method used for their collection. Crucially, the use of biomarkers might expedite and enhance current diagnostic procedures by quickly offering vital clinical insights on the kind, prognosis, response to therapy, and likelihood of recurrence of the cancer, all without requiring tumor tissue specimens. Apart from the HPV indicators, there is an increasing scientific interest in other biomarkers found in bodily fluids, since they might be easily included into clinical practice. Many biomarkers released by cancer cells have been thoroughly investigated for their potential as prognostic, diagnostic, and relapse-predictive indicators [16,17,18,19].

### 2.1. DNA Biomarkers

In recent years, circulating tumor (ct) DNA techniques have been extensively used in many areas of cancer treatment, including screening programs. The increasing limits of cancer detection in blood samples are being pushed by continuous platform and computational technique developments, leading to the emergence of several multi-cancer early detection (MCED) strategies in both clinical and research contexts. Still unknown, nevertheless, is the best time to identify cancer at an advanced stage of development [20,21,22,23].

Early detection techniques based on circulating tumor DNA have great potential to greatly improve cancer-screening initiatives, particularly for malignancies for which there are no screening guidelines. There are just four cancer forms for which population-level screening tests are advised, meaning that many cancer types go undiagnosed until later stages when symptoms start to show [24,25]. The detection of asymptomatic early-stage malignancies creates a unique opportunity to concurrently improve outcomes across these crucial areas, as late-stage cancer diagnosis is associated with lower survival rates, greater healthcare expenditure, and more treatment difficulties [26,27,28]. Viral ctDNA biomarkers are promising in the context of precision medicine, especially in HPV-associated OPSCC [18,19].

**Figure 2:**
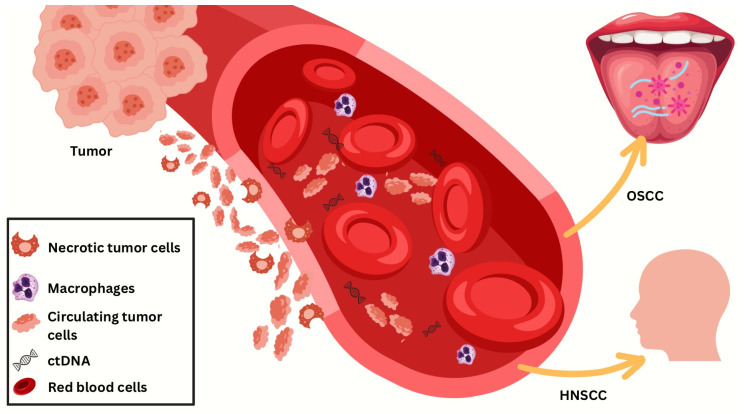
ctDNA as a biomarker in oral and oropharyngeal squamous cell carcinoma. This figure illustrates the circulation of tumor-related materials in the bloodstream relevant to oral squamous cell carcinoma (OSCC) and head and neck squamous cell carcinoma (HNSCC). The tumor releases necrotic tumor cells, macrophages, circulating tumor cells (CTCs), and circulating tumor DNA (ctDNA) into the bloodstream, shown alongside red blood cells. Macrophages as immune cells interact with these tumor components. CTCs are live tumor cells that have shed into the bloodstream, and ctDNA consists of fragments of tumor DNA circulating in the blood. These materials represent various stages of tumor cell degradation and dissemination, with ctDNA a potential cancer detection and monitoring biomarker. The figure highlights the complexity of tumor biology and the potential for blood-based biomarkers in diagnosing and monitoring OSCC and HNSCC.

Cancer cells going through necrosis, apoptosis, or active secretion processes release ctDNA, a subset of circulating free DNA (cfDNA), into the bloodstream [24,25,26,27,28]. The identification of cfDNA in blood-derived materials such as serum or plasma may provide a “liquid biopsy” specimen for cancer surveillance and/or early diagnosis, which may eventually replace solid tumor specimens. Since it rises greatly in sequentially collected samples from patients with HPV-positive OPSCC, the value of ctHPV DNA for early diagnosis, post-treatment monitoring, and recurrence monitoring is rapidly expanding [29] (Figure 2).

In a recent issue of *Clinical Cancer Research*, Berger and colleagues shared significant findings on testing circulating tumor tissue-modified viral (cTTMV) human papillomavirus DNA after treatment for oropharyngeal squamous cell carcinoma (OPSCC) [30]. Additionally, a derivative of Chera and colleagues’ work uses multianalyte digital droplet PCR (ddPCR) to measure circulating cell-free tumor tissue-modified viral DNA (cTTMV DNA). While the assay’s development and specific details interest researchers, clinicians focus more on the outcomes of the test rather than its methodology [18,31].

In the study conducted by Berger et al. mentioned earlier, over 1000 patients who had completed treatment for HPV-related OPSCC underwent more than 1300 cTTMV HPV DNA plasma assays. Of these, 80 (7.4%) tested positive, with 26 already having clinically active cancer. Among the remaining 59 patients, 55 developed recurrent cancer, yielding a positive predictive value (PPV) of 95% and 93% for those without evidence of relapse at the time of testing. Conversely, the negative predictive value (NPV) was 95% among the roughly 1200 negative tests, despite 58 patients having clinically apparent disease at testing time. This unusual NPV endpoint is due to short follow-up and the study’s nature, based on routine clinical care data rather than a prospectively planned trial [30].

Recent data from a phase II chemoradiation study for low-risk HPV-associated OPSCC patients indicated a lower PPV for the TTMV HPV DNA assay. The patients’ outcomes varied significantly by T and N status, and the biomarker’s performance might differ across various treatment modalities, including surgery, radiation, chemoradiation, or immunotherapy [32,33]. In this study, only 37% of patients with positive tests had evidence of progression at two years, and the NPV for progression-free survival (PFS) was 93%.

Currently, post-treatment evaluation for OPSCC includes serial clinical examinations and a single post-treatment imaging scan, with PET-CT recommended three months post-radiation therapy due to its high NPV for relapse [34]. There is limited data supporting the use of subsequent prognostic biomarkers, including PET-CT, for long-term follow-up, which should undergo further studies [35,36,37]. For comparison, the recommendations for nasopharyngeal cancer (NPC), driven by Epstein–Barr virus, suggest plasma EBV DNA monitoring, which correlates with relapse but lacks robust prospective evaluation [38,39]. While the data from Berger et al. suggest that cTTMV HPV DNA could be a valuable prognostic biomarker, more controlled studies are needed to confirm its clinical utility [30]. The high PPV reported needs reconciliation with the modest PPV from other studies before recommending widespread use. Additionally, cost–benefit analyses should be conducted considering the substantial costs of serial testing. There is potential for using cTTMV HPV DNA assays in early detection and treatment response assessments, but further controlled clinical trials are necessary to optimize their use [40].

Saliva has been suggested by some research in identifying particular HPV markers in HNC patients, in addition to biomarkers found in blood-derived specimens [41]. Compared to blood, saliva is a bodily fluid that is easier to collect, yet its lower biomarker concentrations may make it less useful. While oral HPV positivity in healthy people has been documented in several independent studies, a significant incidence of HPV DNA positivity in saliva and gargles has been noted in HPV-related HNSCC patients [42,43,44]. After therapy, oral HPV prevalence and viral load in HNSCC patients tend to decline, although oral HPV that is persistent is linked to recurrence [45].

### 2.2. RNA Biomarkers

The pathophysiology of HPV-positive HNSCC remains incompletely understood, but it is well recognized that the primary oncoproteins of high-risk HPVs, E6 and E7, are essential to the tumorigenesis and development of HPV-positive HNSCC [46]. Moreover, oral HPV mRNA testing has shown sensitivity ranging from 23% to 82% in identifying OPSCCs linked to HPV [44,47].

E6 and E7 can induce mutations and epigenetic changes in the host genome [48]. Additionally, they can deactivate the tumor suppressor proteins p53 and retinoblastoma protein (pRb), which play crucial roles in regulating the cell cycle, maintaining genome stability, and controlling epigenetic modifications [49]. They can also influence the host genome’s mutation and epigenetic modifications [48]. A constantly increasing number of studies have demonstrated that epigenetic modifications also have a major impact on the molecular control of HPV-induced carcinogenesis and advancement [50]. Histone post-translational covalent changes and the impacts of noncoding RNA are examples of epigenetic control that E6 and E7 can induce [51].

#### 2.2.1. MicroRNA

Endogenous noncoding RNAs (ncRNAs) are converted into precursor micro-RNAs (miRNAs) in the nucleus and subsequently transported to the cytoplasm as miRNAs [52]. Through their binding to the 5′- and 3′-UTRs of their target mRNAs, miRNAs have the ability to regulate the production of cellular proteins [52,53]. miRNAs have been primarily linked to transcriptional and translational levels of various phases of malignant transformation of cells [54]. In particular, HPV infection has been linked to the expression level of certain strains of miRNAs. The viral oncoproteins produced by HPV have the ability to alter host gene expression and the number of miRNAs as it integrates into the host genome [55,56]. Numerous studies have looked into certain miRNA expression patterns in HNSCC tissues and cells that are positive for HPV [57]. HPV-positive and HPV-negative samples had different levels of these miRNAs’ expression and regulation. Furthermore, the unique expression of miRNAs in HPV-positive HNSCC considerably influences clinical features in a manner distinct from that of HPV-negative cases. It is important to highlight the different molecular mechanisms for miRNA function that can be observed in HPV-positive and HPV-negative HNSCC.

Diverse techniques for detection have been employed to identify distinct miRNA expression in HPV-positive HNSCC [57,58]. In 51 individuals with OSCC and pharyngeal squamous cell carcinoma (PSCC), differential miRNA expressions were originally described utilizing microarray analysis by Lajer et al. [59,60]. According to their findings, 21 miRNAs were impacted by HPV infection and may cause different clinical traits. In HPV-positive HNSCC, studies have now concentrated on miRNA expression profiles. In HPV-positive OPSCC, Gougousis et al. found that overexpression of miR-15, miR-16, miR-143, miR-145, and the miR-106-363 cluster was seen [58]. Additionally, 30 miRNAs were expressed in HPV-positive samples and 38 in HPV-negative samples in a study of Vojtechova et al., who utilized TaqMan real-time quantitative PCR (RT-PCR) arrays to evaluate differential expression in HPV-positive and -negative tonsillar tumors [61].

Utilizing gene chip and RT-PCR technologies, next-generation sequencing (NGS) has been used for genome differential sequence alignment [62]. miRNA profiles and clinical information about HNSCC are included in the Cancer Genome Atlas (TCGA) data. Variously expressed miRNAs in HPV-positive and -negative HNSCC tissues can be screened using miRNA profiles derived from TCGA data via NGS. In Nunvar et al.’s study, NGS analysis revealed that 70 and 116 distinct miRNAs were differently expressed in HPV-negative and HPV-positive HNSCC, respectively [56].

In HNSCC, variations in miRNAs are useful indicators [63]. In general, miRNAs may function as new biomarkers in HNSCC that is positive for HPV. The overexpression of miR-205-5p, miR-182-5p, and miR-133a-3p in HPV-positive OPSCC was discovered by House et al. and Weiss et al., and these may be used as prognostic indicators [64,65]. According to studies by Gougousis et al. and Bersani et al., miRNAs may be identified between the T2 and T4 stages in HPV-associated tongue squamous cell carcinoma (TSCC) and OPSCC [58,66]. They are also linked to peripheral tumor metastasis, invasion, and migration. Moreover, miR-106a, miR-27a, and miR-9 were strongly related with the sensitivity of HPV-positive HNSCC to radiation, but miR-139-3p was associated with the susceptibility of HPV-positive HNSCC to chemotherapy [55,67].

#### 2.2.2. Mechanism of Action of MicroRNA

As was already noted, miRNAs have been linked to a number of biological processes, which has led scientists to look into their potential regulatory functions in HPV-positive HNSCC [68,69]. As an illustration, Casarotto et al. emphasized the possible importance of miR-139 and miR-375 in regulating the growth of HPV-related HNSCC [68]. Furthermore, Luo et al. provided evidence of the regulatory functions of miR-518a-5p and miR-605-5p in HPV-positive HNSCC cell proliferation, apoptosis, tumor development, and metastatic spread [70]. By focusing on genes and pathways linked to tumor growth, apoptosis, cell proliferation, the epithelial-to-mesenchymal transition (EMT), and secondary cancer formation, these miRNAs serve as essential regulators.

Moreover, miRNAs may be important players in oncogenesis as tumor suppressors or onco-miRNAs [58,71]. In HPV-positive HNSCC, a number of miRNAs, including miR-22, miR-27, miR-92a, miR-195, and miR-211, have been found to function as oncogenic promoters and regulate target genes, which in turn promotes tumorigenesis [72,73]. Research has shown that miR-21 and miR-155 can activate target genes like phosphatase tensin homologue (PTEN) and signal transducer and activator of transcription (STAT), promoting the growth and invasion of OPSCC cells [57]. On the other hand, in HPV-positive HNSCC, miRNAs such as miR-16 and miR-17 have been identified as tumor suppressors. These miRNAs shrink tumors by downregulating certain oncogenes and reviving important tumor suppressor proteins like p53, p21, and p16 [74].

The prognosis of HNSCC may be impacted by immunological responses and cell autophagy according to recent research [75,76]. According to Aranda-Rivera et al., miRNAs have the ability to control immunological responses and cell autophagy, which can affect the outcome of HPV-positive HNSCC [77]. Furthermore, Luo et al. found that in HPV-positive HNSCC, miR-380-5p, miR-338-5p, miR-16-1-3p, and miR-378a-3p activated immune responses, resulting in good prognosis [70]. To gain a deeper understanding of the controlling role and mechanism of miRNAs in HPV-positive HNSCC, further comprehensive research is required.

For patients with HNSCC, radiation therapy is one of the crucial parts of the treatment plan. New research suggests that miRNAs control the radiation response by acting on their target genes [78,79,80]. According to Fu et al. DNA-repair genomic markers can affect how sensitive HPV-positive cancers are to radiation, and miRNAs may be able to modulate DNA damage by focusing on genes downstream [81]. Furthermore, Zhang et al. discovered that via inhibiting RUNX3 and SMG1, respectively, miR-106a and miR-27a increase radiation sensitivity in HPV-induced HNSCC [67,79]. In HPV-negative cells, conversely, overexpression of miR-125b can reduce radiation sensitivity by lowering ICAM2 levels, a protein linked to increased radiosensitivity [57].

#### 2.2.3. Long Noncoding RNA

Long noncoding RNAs (lncRNAs) represent a diverse group of RNA molecules with a length exceeding 200 nucleotides. They do not possess the capacity to encode proteins, and are involved in a number of biological processes through interactions at transcriptional and translational levels with proteins, miRNAs, circRNAs, downstream RNAs, and pseudogenes [57].

Several studies have demonstrated how lncRNAs affect cell invasion, migration, and proliferation, which in turn affects how HNSCC tumors advance [82,83]. Moreover, lncRNAs can operate as competing endogenous RNAs (ceRNAs), ensnaring different RNAs to alter target gene expression, which in turn affects different tumor behaviors [84]. In host cells, HPV infection can cause aberrant lncRNA expression, which can cause downstream molecules in important signaling cascades to become dysregulated [85,86]. There is growing evidence that different lncRNAs can be found in clinical specimens by RT-PCR, RNA-sequencing, and bioinformatic tools [57]. This has led to the cataloguing of aberrant lncRNA expression in HPV-positive HNSCC. Wang et al. discovered 131 lncRNAs that were uniquely expressed in the TCGA HPV-negative HNSCC dataset [87]. Similarly, 140 lncRNA transcripts were discovered that had varying expression levels between HPV-positive and HPV-negative HNSCC [57]. Additionally, as shown by Kopczyńska et al. and Haque et al., HPV infection was linked to altered lncRNA expression [85,88,89,90].

A strong correlation between the expression levels of EGOT in HPV-positive pharyngeal carcinoma and certain clinical features has been found [91]. Additionally, increased PRINS and TTTY15 were found to be positively correlated with a better outcome in HPV-positive HNSCC [85,92]. Moreover, in HNSCC, abnormal lncRNA expression is correlated with its resistance to chemoradiotherapy [57,93]. Lnc-IL17RA-11 levels were discovered to have a substantial association with radiation efficiency in HPV-positive HNSCC by Song et al. in 2019 [93]. On the other hand, lncUCA1 and lncWISP1 were associated with radiation resistance in HPV-negative HNSCC, which shows a wide range of diagnostic possibilities that need to be further examined [94,95].

Certain long noncoding RNAs have been linked to the onset and advancement of HPV-positive HNSCC [96]. The roles of lincRNA-p21, HOTAIR, PROM1, and CCAT1 in tumor formation and metastasis were discovered by Ma et al. and Dias et al. [96,97]. By interacting with miRNAs, these lncRNAs can control the expression of mRNA [83,98,99]. For instance, in OSCC, lncRNA BLACAT1 stimulates invasion and proliferation by sponging miR-142-5p [83]. Nevertheless, little is known about how lncRNAs decrease tumor growth in HPV-positive HNSCC. Based on data published by Sannigrahi et al., MEG3 functions as a tumor suppressor and may induce cellular death by upregulating the target genes IRE1 and GRP78 [100,101]. The survival prognosis of HPV-positive HNSCC is also impacted by abnormal expression of lncRNAs [92,102,103]. According to Guo et al., TTTY15 modifies autophagy-related proteins or processes, which in turn influences prognosis [103]. According to a different study, lncRNA expression controls the immune system’s infiltration into tumors: lower levels of lncRNA may strengthen the immune response and improve prognosis [102]. However, additional clinical information is required to validate these lncRNAs’ prognostic significance in HPV-associated HNSCC.

Like miRNAs, lncRNAs presumably act as molecular sponges to change downstream genes that are important for HPV-positive HNSCC gene stability [57,93]. Therefore, lncRNAs have the ability to greatly increase radiation sensitivity. One example of this is lnc-IL17RA-11, which raises radiation sensitivity by triggering the transcription of estrogen receptor α [93]. On the other hand, radiation resistance is linked to lncUCA1 and lncWISP1 [57].

The 5′-cap and 3′-poly(A) tails of circular noncoding RNA (circRNA), which is produced from precursor messenger RNA (pre-mRNA), are absent [104]. CircRNAs primarily function as molecular sponges, contending with miRNAs or other RNAs to control a range of biological activities [105]. The proliferation, invasion, and spread of tumors are facilitated by dysregulated circRNA expression [106]. A possible association between the expression of circRNA and oncoprotein in HPV-positive malignancies was presented by Tornesello et al. [107]. Research conducted by Chen et al. and Jun et al. demonstrated unique patterns of circRNA expression in OSCC, a cancer that frequently exhibits elevated rates of HPV infection [108,109]. Additionally, in OSCC, Bonelli et al. linked circRNAs to carcinogenesis, cancer spread, and resistance to treatment. Several of these relationships were also connected to TNM staging [110].

Additionally, circRNAs affect the clinical characteristics of OSCC. Zhao et al. found prognostic markers by correlating particular circRNAs with tumor grade and TNM stage [111]. A study by Cristóbal et al. connected some circRNAs to the migration and proliferation of cancer in OSCC, whereas other circRNAs were connected to chemotherapy resistance [112,113]. Through sponging miRNAs and influencing target genes downstream, circRNAs seem to control the development of tumors [110]. Further research is necessary to fully comprehend the role of circRNA in HPV-positive HNSCC and how it regulates viral oncoproteins.

PiRNA interacts with PIWI proteins to contribute to gene silence. PiRNA is a form of noncoding RNA that is roughly 26–30 nucleotides long [114]. PiRNA expression may be influenced by HPV status: in HPV-positive HNSCC, specific piRNAs have been found to be prognostic indicators [115,116]. Notably, Firmino et al. related certain piRNAs to oncogenesis, whereas Krishnan et al. linked others to pathological stage and nodal metastasis. PIWI proteins are frequently the target of these piRNAs’ interactions, which control carcinogenesis and cell division [115,117]. Nevertheless, little is known about their regulation or interaction with viral oncoproteins in HPV-positive HNSCC.

Mostly involved in RNA production and modification, snoRNA is a kind of noncoding RNA that ranges in length from 60 to 300 nucleotides [118]. Clinical characteristics of HNSCC were found to be associated with snoRNA expression by Xing et al., indicating that these markers may be useful for tracking the development of tumors. Tumor progression, histological grade, and clinical staging have all been linked to certain snoRNAs [119]. The connection between snoRNAs and HPV infection in HNSCC, as well as their potential regulatory function in HPV-positive HNSCC, is still mainly unknown. To fully understand their role in carcinogenesis and clinical traits, more investigation is required.

### 2.3. Protein Biomarkers

In patients with oropharyngeal squamous cell carcinoma, it is advised that testing for HPV using p16 immunohistochemistry (IHC) be conducted, with optional molecular HPV-DNA testing added [119,120]. Although p16 IHC is a very sensitive surrogate marker for HPV-related OPSCC, there has not been as much research on its application for other kinds of HNSCC [121,122]. Various HPV testing techniques are used in addition to p16 IHC, frequently in combination with p16INK4a IHC [123]. When it comes to identifying HPV in HNSCC, E6 oncoprotein testing—like OncoE6TM—has shown encouraging sensitivity and specificity. For example, Menegaldo et al. found that OncoE6TM had significant sensitivity and specificity in detecting HPV16/18 E6 oncoproteins [124]. Similar to this, Chernesky et al. evaluated the presence of oncoproteins, HPV E6, and nucleic acids in samples collected from individuals with OPSCC, noting varying degrees of agreement with other HPV markers and p16 antigen staining [125].

As described by Agustin et al. several HPV testing methods have different benefits and drawbacks [126]. For example, p16 IHC is a feasible and affordable diagnostic technique, with sensitivity and specificity ranging from 80% to 90% [127]. High sensitivity but limited specificity are the characteristics of DNA PCR techniques, despite their stability and reproducibility. HPV mRNA E6/E7 RT PCR provides excellent sensitivity and specificity, but its routine use is limited by the need for fresh or frozen material and technical expertise [123,126]. HPV DNA ISH reduces the possibility of false-positive results due to tissue contamination by allowing direct observation of the virus inside tumor cells [123]. Novel biomarkers for the treatment of HPV-related OPSCC have been investigated in recent studies. Antibodies against the E6 protein, for example, have been linked to a significantly elevated risk of OPSCC, frequently manifesting more than 10 years prior to diagnosis. Although these antibodies are uncommon in healthy people, HPV-positive OPSCC patients frequently have them, especially if their tumors are connected to HPV16. While some researchers suggest employing E6 serology to monitor HPV OPSCC, particularly for recurrence or persistent disease, more study and validation are required before practical application [126].

Apart from E6 antibodies, as mentioned before, ctDNA from plasma has become popular in HPV-related cervical squamous cell carcinoma (CSCC) and HNSCC when identified by ultra-sensitive droplet digital PCR [19,128]. High sensitivity and specificity of ddPCR in detecting HPV16 and HPV33 subtypes in OPSCC plasma indicate possible uses in disease response monitoring and early detection screening [19]. Furthermore, based on residual HPV ctDNA levels after therapy, ddPCR-based HPV ctDNA detection may help predict relapse in CSCC [128]. When it comes to the detection of cervical neoplasia at different phases of the disease, ddPCR is more advantageous than RT-PCR because it provides more accurate and sensitive measurement of low target DNA quantities [129].

Since ddPCR is quantitative in nature and has good sensitivity, precision, and repeatability, it is a promising method for detecting HPV biomarkers [29,126]. ddPCR is particularly advantageous in clinical settings, since it is affordable and can identify samples with low DNA concentrations, like swabs [126].

#### Utilizing Droplet Digital PCR in HPV Diagnostics

By encasing the target nucleic acid molecules in discrete, precisely defined water-in-oil droplet partitions, ddPCR measures the absolute number of these molecules [127,130]. In ddPCR, templates are diluted to single-molecule levels and DNA molecules are counted using Poisson distribution assumptions [131]. Sample preparation is conducted in smaller, precisely measured portions or partitions, each of which is processed separately. It is similar to traditional PCR reactions using TaqMan hydrolysis probes or DNA-binding dyes. Using Poisson distribution, positive reactions are found and measured inside each division [131,132]. The ddPCR system consists of three main parts. First is droplet generation, meaning the usage of a droplet generator to divide samples into 20,000 uniform nanoliter-sized droplets for accurate target amplification. Second is amplification, in which heating the droplets leads to the amplified PCR by annealing, extending, and denaturing them, and the third part is droplet reading. This is carried out using a droplet reader to measure each droplet’s fluorescence in two channels [133].

ddPCR is especially useful for HPV detection in OPSCC because it provides excellent sensitivity, specificity, and accurate quantification for detecting target DNA. Studies have measured viral load (VL) and detected HPV DNA in CSCC using ddPCR. VL is a key factor in determining the persistence of the virus and is used as a biomarker for prognosis assessment and treatment response monitoring in HPV-related disorders [132,134]. ddPCR has been used in CSCC to identify HPV in a variety of sample types, including cell lines, formalin-fixed paraffin-embedded (FFPE) tissues, and cervical liquid cytology samples. With no difference in VL between tumors with numerous and single HPV infections, Malin et al. showed the great sensitivity of ddPCR in detecting HPV and VL in FFPE tissues and cervical liquid cytology samples [132]. The ability of ddPCR to measure larger copy numbers than qRT-PCR was highlighted by Larsson et al. when they compared the two methods for measuring HPV VL in FFPE tissues and liquid-based cytology samples [134]. Rotondo et al. used ddPCR to measure HPV DNA in human cell lines and CIN tissues, demonstrating its accuracy in identifying and measuring several HPV strains at once [135].

## 3. Tissue-Based Biomarkers

One well-known proxy sign for oncogenic HPV infection is the overexpression of p16. The 8th edition of the *AJCC Cancer Staging Manual* included specific staging for p16-positive OPSCC due to its correlation with better survival rates in HPV-positive patients [136,137]. With a sample of 237 OPSCC patients, Barber et al. showed that p16 is an independent predictor of improved recurrence-free survival and is associated with nodal disease at presentation [138].

The lungs are a typical location for second primary tumors and host the majority of distant metastases from HNSCC. It might be difficult to distinguish between a second primary lung SCC and HNSCC metastases. In HPV-related OPSCC, distant metastases maintain their p16 and HPV status, with constant transcriptional activity for HPV E6 and E7 oncoproteins according to Mehrad et al. [139]. In order to distinguish between HNSCC metastasis and second primary lung SCC, they advise conducting HPV testing using PCR or DNA in situ hybridization in addition to morphological comparison.

A minimally invasive diagnostic technique called fine-needle aspiration (FNA) can be used to assess suspicious neck masses. According to Chernesky et al., FNA-obtained cells may be evaluated for malignancy and HPV E6 oncoproteins. HPV-positive HNSCC frequently metastasizes to cervical lymph nodes. With agreements of 81.4%, 94.9%, and 91.1%, respectively, they discovered good agreement rates for p16 antigen testing of primary oropharyngeal tumors with FNA-tested E6 oncoproteins, HPV E6/E7 mRNA, and HPV DNA [125].

Cervical lesions may be screened for both malignant and pre-malignant lesions using a variety of completely developed and tested procedures and algorithms. Independent studies have proposed a number of tests for HPV-positive HNSCC, but they need to be verified in screening programs. Indirect/surrogate HPV indicators, including p16INK4a staining, viral DNA detection by PCR or in situ hybridization (ISH), and the identification of E6/E7 mRNA by RT-PCR or RT-qPCR, can all be used to distinguish between HPV-driven and non-HPV-driven HNSCC. PCR-based techniques for HPV DNA identification in cancer tissue show excellent sensitivity but reduced specificity because of the potential for transitory infections in the upper respiratory and digestive tracts [140]. While ISH for HPV DNA offers details on the location of the virus within cells, it is not as sensitive as PCR-based tests [141]. Finding full-length E6/E7 mRNA in cancer tissue—which can be accomplished using FFPE material—is the gold standard for proving HPV’s carcinogenic significance [141,142]. As an alternative, it has been confirmed that the spliced form of the E6 gene, or E6*I mRNA, may be used as a marker to categorize HR HPV-positive HNSCCs [143]. However, the requirement for specialized facilities may limit the application of HPV mRNA detection techniques in clinical application.

### Optimizing HPV Biomarker Algorithms for Accurate OPSCC Prognosis

Compared to HPV-negative OPSCCs, it seems that HPV-induced OPSCCs have a better prognosis [144]. Thus, several studies have focused on developing algorithms for quick HNSCC categorization. As mentioned earlier, the specificity of an algorithm is greatly increased when many HPV markers are used, while maintaining sensitivity [145]. In order to achieve 98–100% sensitivity and specificity in oral cavity and oropharynx FFPE samples, Smeets and colleagues developed an algorithm that integrated the use of p16INK4a and HPV DNA. On the other hand, p16NK4a sensitivity was 100% but specificity was only 85% when DNA ISH was used in conjunction with it in HNSCCs [141].

Combining p16INK4a with HPV DNA PCR produced 93% sensitivity and 96% specificity for viral mRNA-positive OPSCCs, a sensitivity that was not considerably lower but noticeably more specific than separate tests, according to a meta-analysis by Prigge et al. that included 24 investigations [123].

While p16INK4a staining in combination with other HPV tests is a legitimate method for HNSCC categorization, proper interpretation of p16INK4a staining requires care. Moreover, in certain HNSCCs, additional risk factors may have an impact on p16INK4a expression, which might explain differences in p16INK4a expression and HPV DNA PCR positivity [141,146].

p16INK4a and HPV DNA PCR have been assessed in several investigations in addition to E6/E7 mRNA detection [147,148]. The HPV-attributable proportion was shown to be reduced in FFPE tissues when E6 mRNA, HPV DNA PCR, and p16INK4a were detected together. It was noted in an Indian investigation that additional risk factors had an impact on p16INK4a expression in this location, since almost 60% of HNSCC specimens testing HPV DNA/mRNA-double-positive were p16INK4a-negative and approximately 18% of HPV mRNA-negative HNSCCs were p16INK4a-positive [149]. Hence, integrating triage with indirect cellular indicators such as p16INK4a and direct HPV markers like HR HPV DNA detection complemented with mRNA could enhance the categorization of HPV-related HNSCC. In cases where there is a disparity among HPV markers (e.g., HPV DNA−/p16+), the analysis of mRNA aids in identifying clinically significant HPV infections. The use of HPV DNA testing has the potential to enhance the specificity of p16INKa labeling.

## 4. Emerging Technologies Revolutionizing HPV-Positive HNSCC Diagnosis

### 4.1. CRISPR-Based Diagnostic Tools

Clustered regularly interspaced short palindromic repeat (CRISPR)-based diagnostic instruments have surfaced as a groundbreaking method for the identification and treatment of numerous illnesses, such as OPSCC that is positive for HPV. Utilizing the accuracy and effectiveness of CRISPR technology—particularly the CRISPR-Cas systems—these instruments pinpoint precise genomic sequences linked to pathogens or mutations that cause disease [150,151].

CRISPR-based diagnostics provide a number of benefits over conventional techniques like PCR and immunohistochemistry, which frequently have drawbacks concerning sensitivity, specificity, and the speed at which viral loads can be quickly determined in the case of HPV-positive OPSCC [152]. For early diagnosis and tracking the course of the disease, CRISPR diagnostics can detect HPV DNA with high precision and at reduced virus levels. The SHERLOCK platform, which stands for “specific high-sensitivity enzymatic reporter unlocking”, is a CRISPR diagnostic tool [153,154].

The Cas13 enzyme, which SHERLOCK uses, binds to its target RNA to initiate collateral cleavage activity that cleaves neighboring RNA molecules and generates a detectable signal [155,156]. Three key phases are involved in the mechanism of CRISPR. The first is target recognition. The HPV DNA sequence-specific guide RNA (gRNA) is used to program the CRISPR–Cas13 complex. The Cas13 enzyme is directed to bind to the target sequence by the gRNA when the sample contains HPV DNA [150]. The second phase is known as “activation and cleavage”. When Cas13 binds to the HPV DNA target, it changes its conformation, which initiates the process of collateral cleavage [157,158]. This causes the nearby RNA molecules that are labeled with fluorescent or colorimetric reporters to cleave non-specifically [150,158]. Finally, in the third step, signal detection is when these reporter molecules cleave: a discernible signal—like fluorescence or a change in color—occurs, signaling that HPV DNA is present in the sample. Due to its excellent sensitivity, this procedure can identify very low amounts of HPV DNA, which is essential for early diagnosis and surveillance [150].

The DETECTR (DNA endonuclease-targeted CRISPR trans reporter) system is an additional sophisticated CRISPR diagnostic tool. The Cas12 enzyme used by DETECTR is directed to the target DNA sequence by a gRNA, just as with Cas13. In addition to its cleavage activity upon binding, Cas12 also targets single-stranded DNA (ssDNA) reporters. Similar phases for target recognition, activation, and signal detection are followed by the mechanism, which offers a flexible and extremely sensitive way to detect HPV in OPSCC [159,160].

Portable point-of-care devices can be integrated with CRISPR-based diagnostics, enabling their use in a variety of clinical contexts. This is especially helpful for OPSCC patients who are HPV-positive, as prompt and precise identification is necessary for efficient treatment planning [161]. Compared to conventional approaches, CRISPR diagnostics have a speedy turnaround—often within an hour—that facilitates faster clinical decision-making. Additionally, multiplexed detection—the simultaneous identification of numerous HPV genotypes or other pertinent indicators in a single test—is made possible by CRISPR diagnostics [162]. Understanding the entire viral landscape in OPSCC patients is important because it will help develop more specialized and focused treatment plans [163].

In HPV-positive OPSCC, CRISPR-based diagnostics play a more significant role than just detection [164]. Through the monitoring of viral load both before and after therapy, these technologies can identify possible recurrences sooner than traditional methods and offer valuable insights into the success of the treatment. The capacity to monitor continuously is essential for controlling OPSCC, as early relapse diagnosis can have a major impact on prognosis [165].

### 4.2. Next-Generation Sequencing in HPV-Related Diagnostics

Since next-generation sequencing (NGS) offers high-throughput capabilities and comprehensive genomic insights, it has completely changed the diagnostics of HPV-related OPSCC [166]. Understanding the molecular mechanisms behind HPV-driven carcinogenesis requires an in-depth examination of the HPV genome and its integration into host DNA, which is made possible by the simultaneous sequencing of millions of DNA fragments made possible by NGS [167]. When it comes to HPV-related OPSCC, NGS is superior to conventional diagnostic techniques like PCR and immunohistochemistry in a number of ways. The ability of NGS to distinguish between distinct HPV genotypes is crucial, since different HPV types are linked to differing carcinogenic potentials and clinical outcomes. Planning individualized treatments and risk stratification are made easier by this accurate genotyping [167,168].

The detection of HPV DNA and RNA is a major advantage of NGS in HPV-related OPSCC diagnostics. Viral load can be measured with NGS, giving information about the degree of infection and how it relates to the severity of the disease. Moreover, NGS can identify the expression of viral oncogenes E6 and E7, which are important initiators of oncogenesis in HPV-positive malignancies, by examining viral RNA. The confirmation of HPV causal function in the tumor is aided by this expression profiling [169].

Finding the genomic changes that HPV incorporation has caused in the host genome is another important use. With the help of NGS, one can uncover structural differences, copy-number variations, and mutations resulting from viral integration, providing a comprehensive picture of the genomic alterations linked to HPV-driven transformation [170,171]. Moreover, NGS makes it possible to analyze the transcriptome and immune cell infiltration of the tumor, which further advances our understanding of the tumor microenvironment. By identifying the immune evasion mechanisms used by HPV-driven malignancies, this thorough profiling can help guide immunotherapeutic approaches [172]. Additionally, NGS is essential for tracking the course of a disease and the effectiveness of treatment. Clinicians can identify early indications of recurrence and minimal residual disease by comparing successive samples, which enables prompt intervention and better patient outcomes [173].

### 4.3. Nanodiagnostics and Biosensor Technologies

Traditional diagnostic methods in OPSCC include histopathological examination, imaging, and molecular testing, which, while effective, have limitations in terms of sensitivity, specificity, and early detection capabilities. The field of HPV-positive oropharyngeal cancer diagnosis and therapy is quickly changing due to advancements in biosensor and nanodiagnostic technology. Advances in nanodiagnostics and biosensor technologies offer promising alternatives that enhance diagnostic accuracy and enable earlier detection. Nanodiagnostics influences nanotechnology for disease diagnosis at the molecular level. This approach involves the use of nanoparticles, quantum dots, nanoshells, and nanotubes to improve the detection of biomarkers associated with HPV and OPSCC. These cutting-edge technologies greatly improve early diagnosis and individualized treatment plans by providing highly sensitive, precise, and quick detection of HPV infections and associated oncogenic indicators [174].

Utilizing nanomaterials and nanotechnology, nanodiagnostics aims to enhance the identification of cellular and molecular indicators linked to HPV-positive OPSCC. The creation of assays based on nanoparticles, which are highly accurate in detecting HPV DNA or RNA, is one well-known use. By precisely binding to HPV genetic material, these nanoparticles can be designed to enable the accurate and efficient detection of viral presence, even at low concentrations [175]. Examples of nanomaterials used to magnify detection signals include magnetic, gold, and quantum dots. These materials offer ultra-sensitive diagnostic capabilities that outperform conventional techniques like PCR. Nanoparticles (NPs) can be engineered to bind specifically to HPV DNA/RNA or proteins expressed in OPSCC. Gold nanoparticles (AuNPs) and magnetic nanoparticles (MNPs) are particularly useful due to their unique optical and magnetic properties, respectively. For instance, AuNPs conjugated with antibodies against HPV-related proteins can be used in surface plasmon resonance (SPR) assays for highly sensitive detection. In addition, quantum dots (QDs) are semiconductor NPs that exhibit unique optical properties such as size-tunable light emission. QDs conjugated with molecular probes can be employed to detect HPV DNA or RNA sequences with high sensitivity and specificity through fluorescence resonance energy transfer (FRET) assays. Moreover, nanoshells and nanotubes can enhance signal detection in various assays. Nanoshells can be used in combination with Raman spectroscopy to detect molecular signatures of HPV and cancer biomarkers. Carbon nanotubes (CNTs) can serve as highly sensitive electrochemical sensors for detecting biomolecules at very low concentrations [176]. Nanodiagnostics can provide detailed molecular profiles of tumors, facilitating personalized treatment strategies based on the specific characteristics of the patient’s cancer [177].

By offering systems that combine biological recognition components with tangible transducers to transform biological interactions into quantifiable signals, biosensor technologies supplement nanodiagnostics. Biosensors (electrochemical, optical, and microfluidic) are analytical devices that combine a biological sensing element with a physicochemical transducer to detect specific analytes, and their advantage is that they can be designed for rapid and accurate diagnostics. In the context of HPV and HNC, biosensors can detect viral DNA/RNA, proteins, and other biomarkers with high precision. Biosensors are able to identify biomarkers that are essential for HPV-driven carcinogenesis, such as E6 and E7 oncoproteins, in the setting of HPV-positive OPSCC and can be used for monitoring treatment response and disease recurrence by detecting changes in biomarker levels over time [178].

For the management of HPV-positive OPSCC, the combination of biosensor and nanodiagnostic technologies offers a number of benefits. These include the possibility of point-of-care testing, quick turnaround times, and high sensitivity and specificity. This is especially helpful in environments with limited resources where traditional laboratory infrastructure could be absent. Enhanced sensitivity of nanodiagnostic tools allows for the detection of HPV and cancer biomarkers at earlier stages, potentially leading to earlier intervention and improved patient outcomes. Additionally, real-time biomarker level monitoring can help with early recurrence detection and treatment response assessment, both of which can improve patient outcomes [175,176,177,178].

## 5. Biomarker-Targeted Treatment Strategies in HPV-Positive and HPV-Negative HNSCC

HPV-positive and HPV-negative HNSCC have distinct etiologies, molecular profiles, and clinical behaviors. HPV-positive HNSCC, often found in the oropharynx, is primarily caused by infection with high-risk HPV types, particularly HPV-16, with sexual behavior being a significant risk factor. These cancers are more common in younger, nonsmoking individuals. In contrast, HPV-negative HNSCC is commonly associated with tobacco and alcohol use, usually in older patients [1,2,3,4].

Diagnosing HPV-positive HNSCC involves detecting HPV DNA or RNA in tumor tissues, often using PCR-based assays or in situ hybridization. Immunohistochemical staining for p16, a surrogate marker for HPV infection, is commonly used due to its practicality and cost-effectiveness. However, distinguishing between causal HPV infection and incidental presence remains a challenge. Diagnosing HPV-negative HNSCC relies on histopathological examination and identifying characteristic genetic mutations. The absence of a viral marker like HPV complicates early detection, and the high molecular heterogeneity poses challenges for accurate diagnosis and effective treatment stratification. These differences necessitate varied approaches to diagnosis and treatments [1,2,3,4,179].

Concerning molecular and genetic profiles, HPV-positive HNSCC is characterized by the expression of viral oncoproteins E6 and E7, which inactivate tumor suppressor proteins p53 and Rb, respectively. This leads to genomic instability and dysregulation of cell cycle control. On the other hand, HPV-negative HNSCC often exhibits mutations in TP53, CDKN2A (p16), and other genes involved in cell cycle regulation and DNA repair. These cancers tend to have a higher mutational burden compared to HPV-positive tumors. Patients with HPV-positive HNSCC often present with smaller primary tumors but larger and more cystic lymph node metastases. They generally have a better prognosis and respond more favorably to treatment. On the other hand, patients affected by HPV-negative HNSCC typically present with more extensive primary tumors and a higher likelihood of locoregional recurrence. The prognosis is generally poorer compared to HPV-positive cases [1,2,3,4,179,180].

Despite a better prognosis for HPV-positive HNSCC, the optimal extent of treatment deintensification remains uncertain, and there is a need for reliable biomarkers to guide therapy decisions. Moreover, the development of resistance to ICIs is a concern that necessitates ongoing research. On the other hand, the poor prognosis and high recurrence rates of HPV-negative HNSCC highlight the need for novel therapeutic strategies. The molecular diversity of HPV-negative tumors complicates the development of universally effective targeted therapies. Additionally, managing the side effects of aggressive treatment regimens is a significant challenge [179,180].

Biomarker-targeted therapies for HNSCC focus on exploiting specific molecular abnormalities present in the tumors. Given the molecular and clinical differences of HPV-positive and HPV-negative HNSCC, biomarker-targeted treatment strategies are challenging. The expression of viral antigens (E6 and E7) makes HPV-positive tumors more immunogenic. Immune checkpoint inhibitors (ICIs), such as pembrolizumab and nivolumab, which target PD-1/PD-L1 pathways, have shown efficacy in treating HPV-positive HNSCC by enhancing the immune response against cancer cells [181]. In addition, vaccines targeting HPV oncoproteins aim to provoke a robust immune response. Therapeutic vaccines, like HPV E6/E7 peptide-based vaccines, are in clinical trials and hold promise for controlling or eradicating HPV-positive tumors [182]. Strategies in HPV-negative HNSCC include epidermal growth factor receptor (EGFR) inhibitors, since the overexpression of EGFR is common in HPV-negative HNSCC. Cetuximab, an anti-EGFR monoclonal antibody, is approved for use in these cancers, either alone or in combination with radiation or chemotherapy [183]. In addition, small-molecule inhibitors targeting specific mutations (e.g., PI3K–Akt–mTOR pathway inhibitors) are being investigated in clinical trials. These therapies aim to disrupt aberrant signaling pathways driving cancer growth. Predictive biomarkers play a crucial role in optimizing biological therapies for HNSCC. By identifying patients most likely to benefit from specific treatments, these biomarkers enable personalized therapy, improving outcomes and minimizing unnecessary toxicity. Finally, combination therapies combining targeted therapies with conventional treatments (chemotherapy, radiation) or immunotherapies may improve outcomes [184,185].

## 6. Conclusions

The distinct molecular and clinical landscapes of HPV-positive and HPV-negative HNSCC demand tailored diagnostic and therapeutic approaches. Advances in biomarker-targeted treatments offer hope for improved outcomes, but significant challenges remain in diagnosing and managing these cancers. The investigation of biomarkers in HPV-positive HNSCC is a move towards precision medicine. Definitive diagnosis of HPV-positive HNSCC is required to establish a model incorporating the molecular characteristics, immune microenvironment, and clinical outcomes of these patients, revealing the potential for HPV status in prognosis and treatment considerations. New opportunities have arisen from better identification of DNA, RNA, and protein biomarkers in bodily fluids and tissues for minimally or non-invasive cancer diagnosis, surveillance, and response to therapy.

Several ctDNA and specific biomarkers have been found, such as p16, E6/E7 oncoproteins, and different noncoding RNAs, including microRNAs and long noncoding RNAs, to help in diagnostics, prognostics, and management. ddPCR, NGS, and novel CRISPR-based diagnostics, for example, have shown remarkable sensitivity and specificity in detecting HPV-related cancers.

In the future, different large-scale clinical trials need to be conducted to validate the performance of such biomarkers in real-world patient populations. A comprehensive multi-omics type of dataset, including genomics, transcriptomics, proteomics, and epigenomics, will play a crucial role in unveiling intricate molecular mechanisms of HPV-positive HNSCC. Moreover, there is an urgent need for the advancement of diagnostic tests that are simple and cheap to use, notably in settings where the resources are poor. That will involve sorting out non-invasive methods, including saliva-based HPV detection.

In addition, personalized treatment strategies targeting patient-specific biomarker profiles will enhance therapeutic efficacy and reduce toxicities, and the implementation of routine surveillance strategies utilizing established tumor biomarkers will be indispensable for the early detection of recurrence and to better manage acquired treatment resistance. Exploring and validating additional biomarkers in immune responses and cell autophagy will improve prognostic predictions and therapeutic targets. Interdisciplinary collaborations are necessary for the research, clinical, and bioinformatic teams to learn and integrate findings coming from all the studies and make better clinical intervention. In acknowledging these steps, the promise of precision medicine in the management of HPV-positive HNSCC can be realized through increased potential for biomarkers and favorable patient outcomes.

## Data Availability

No new data were created or analyzed in this study. Data sharing is not applicable to this article.

## References

[1] Bhat G.R., Hyole R.G., Li J. (2021). Head and neck cancer: Current challenges and future perspectives. Adv. Cancer Res..

[2] Chow L.Q.M. (2020). Head and neck cancer. N. Engl. J. Med..

[3] Gormley M., Creaney G., Schache A., Ingarfield K., Conway D.I. (2022). Reviewing the Epidemiology of Head and Neck Cancer: Definitions, Trends and Risk Factors. Br. Dent. J..

[4] Rettig E.M., Sethi R.K.V. (2021). Cancer of the Oropharynx and the Association with Human Papillomavirus. Hematol./Oncol. Clin. N. Am..

[5] Zhang M., Luo Z., Liu S., Zhang X., Wang L., Wang C. (2024). Identification of an 8 HPV-related RNA signature as a novel prognostic biomarker for squamous cell carcinoma of the head and neck. Medicine.

[6] Leemans C.R., Snijders P.J.F., Brakenhoff R.H. (2018). The molecular landscape of head and neck cancer. Nat. Rev. Cancer.

[7] Oton-Gonzalez L., Rotondo J.C., Lanzillotti C., Mazzoni E., Bononi I., Iaquinta M.R., Cerritelli L., Malagutti N., Ciorba A., Bianchini C. (2021). Serum HPV16 E7 oncoprotein is a recurrence marker of oropharyngeal squamous cell carcinomas. Cancers.

[8] Qian X., Nguyen D.T., Dong Y., Sinikovic B., Kaufmann A.M., Myers J.N., Albers A.E., Graviss E.A. (2019). Prognostic score predicts survival in HPV-negative head and neck squamous cell cancer patients. Int. J. Biol. Sci..

[9] Eberly H.W., Sciscent B.Y., Lorenz F.J., Rettig E.M., Goyal N. (2024). Current and Emerging Diagnostic, Prognostic, and Predictive Biomarkers in Head and Neck Cancer. Biomedicines.

[10] Ludwig S., Sharma P., Wise P., Sposto R., Hollingshead D., Lamb J., Lang S., Fabbri M., Whiteside T.L. (2020). mRNA and miRNA profiles of exosomes from cultured tumor cells reveal biomarkers specific for HPV16-positive and HPV16-negative head and neck cancer. Int. J. Mol. Sci..

[11] Ferris R.L., Spanos W.C., Leidner R., Gonçalves A., Martens U.M., Kyi C., Sharfman W., Chung C.H., Devriese L.A., Gauthier H. (2021). Neoadjuvant nivolumab for patients with resectable HPV-positive and HPV-negative squamous cell carcinomas of the head and neck in the checkmate 358 trial. J. Immunother. Cancer.

[12] Welters M.J.P., Ma W., Santegoets S.J.A.M., Goedemans R., Ehsan I., Jordanova E.S., van Ham V.J., van Unen V., Koning F., van Egmond S.I. (2018). Intratumoral HPV16-specific T cells constitute a type I-oriented tumor microenvironment to improve survival in HPV16-driven oropharyngeal cancer. Clin. Cancer Res..

[13] Bosetti C., Carioli G., Santucci C., Bertuccio P., Gallus S., Garavello W., Negri E., La Vecchia C. (2020). Global Trends in Oral and Pharyngeal Cancer Incidence and Mortality. Int. J. Cancer.

[14] Conway D.I., Purkayastha M., Chestnutt I.G. (2018). The Changing Epidemiology of Oral Cancer: Definitions, Trends, and Risk Factors. Br. Dent. J..

[15] Fakhry C., Krapcho M., Eisele D.W., D’Souza G. (2018). Head and Neck Squamous Cell Cancers in the United States Are Rare and the Risk Now Is Higher among White Individuals Compared with Black Individuals. Cancer.

[16] Arantes L.M.R.B., De Carvalho A.C., Melendez M.E., Lopes Carvalho A. (2018). Serum, plasma and saliva biomarkers for head and neck cancer. Expert Rev. Mol. Diagn..

[17] Kaczor-Urbanowicz K.E., Wei F., Rao S.L., Kim J., Shin H., Cheng J., Tu M., Wong D.T.W., Kim Y. (2019). Clinical validity of saliva and novel technology for cancer detection. Biochim. Biophys. Acta, Rev. Cancer.

[18] Chera B.S., Kumar S., Beaty B.T., Marron D., Jefferys S., Green R., Goldman E.C., Amdur R., Sheets N., Dagan R. (2019). Rapid clearance profile of plasma circulating tumor HPV type 16 DNA during chemoradiotherapy correlates with disease control in HPV-associated oropharyngeal cancer. Clin. Cancer Res. Off. J. Am. Assoc. Cancer Res..

[19] Damerla R.R., Lee N.Y., You D., Soni R., Shah R., Reyngold M., Katabi N., Wu V., McBride S.M., Tsai C.J. (2019). Detection of early human papillomavirus-associated cancers by liquid biopsy. JCO Precis. Oncol..

[20] Chen X., Gole J., Gore A., He Q., Lu M., Min J., Yuan Z., Yang X., Jiang Y., Zhang T. (2020). Non-invasive early detection of cancer four years before conventional diagnosis using a blood test. Nat. Commun..

[21] Lennon A.M., Buchanan A.H., Kinde I., Warren A., Honushefsky A., Cohain A.T., Ledbetter D.H., Sanfilippo F., Sheridan K., Rosica D. (2020). Feasibility of blood testing combined with PET-CT to screen for cancer and guide intervention. Science.

[22] Liu M.C., Oxnard G.R., Klein E.A., Swanton C., Seiden M.V. (2020). Sensitive and specific multi-cancer detection and localization using methylation signatures in cell-free DNA. Ann. Oncol. Off. J. Eur. Soc. Med. Oncol..

[23] Gydush G., Nguyen E., Bae J.H., Blewett T., Rhoades J., Reed S.C., Shea D., Xiong K., Liu R., Yu F. (2022). Massively parallel enrichment of low-frequency alleles enables duplex sequencing at low depth. Nat. Biomed. Eng..

[24] U.S. Preventive Services Task Force, Grade A and B, Category Cancer. https://uspreventiveservicestaskforce.org/uspstf/topic_search_results?topic_status=All&grades%5B%5D=A&grades%5B%5D=B&category%5B%5D=15&searchterm.

[25] Sung H., Ferlay J., Siegel R.L., Laversanne M., Soerjomataram I., Jemal A., Bray F. (2021). Global Cancer Statistics 2020: GLOBOCAN Estimates of Incidence and Mortality Worldwide for 36 Cancers in 185 Countries. CA Cancer J. Clin..

[26] Hubbell E., Clarke C.A., Aravanis A.M., Berg C.D. (2021). Modeled Reductions in Late-stage Cancer with a Multi-Cancer Early Detection Test. Cancer Epidemiol. Biomark. Prev..

[27] Siegel R.L., Miller K.D., Wagle N.S., Jemal A. (2023). Cancer statistics, 2023. CA Cancer J. Clin..

[28] Clarke C.A., Hubbell E., Kurian A.W., Colditz G.A., Hartman A.R., Gomez S.L. (2020). Projected Reductions in Absolute Cancer-Related Deaths from Diagnosing Cancers Before Metastasis, 2006–2015. Cancer Epidemiol. Biomark. Prev..

[29] Veyer D., Wack M., Mandavit M., Garrigou S., Hans S., Bonfils P., Tartour E., Bélec L., Wang-Renault S.F., Laurent-Puig P. (2020). HPV circulating tumoral DNA quantification by droplet-based digital PCR: A promising predictive and prognostic biomarker for HPV-associated oropharyngeal cancers. Int. J. Cancer.

[30] Berger B.M., Hanna G.J., Posner M.R., Genden E.M., Lautersztain J., Naber S.P., Del Vecchio Fitz C., Kuperwasser C. (2022). Detection of occult recurrence using circulating tumor tissue modified viral HPV DNA among patients treated for HPV-driven oropharyngeal carcinoma. Clin. Cancer Res..

[31] Chera B.S., Kumar S., Shen C., Amdur R., Dagan R., Green R., Goldman E., Weiss J., Grilley-Olson J., Patel S. (2020). Plasma circulating tumor HPV DNA for the surveillance of cancer recurrence in HPV-associated oropharyngeal cancer. J. Clin. Oncol..

[32] Yom S.S., Torres-Saavedra P., Caudell J.J., Waldron J.N., Gillison M.L., Xia P., Truong M.T., Kong C., Jordan R., Subramaniam R.M. (2021). Reduced-dose radiation therapy for HPV-associated oropharyngeal carcinoma (NRG Oncology HN002). J. Clin. Oncol..

[33] Yom S.S., Torres-Saavedra P.A., Kuperwasser C., Kumar S., Gupta P.B., Ha P., Cao H., Lee N.Y., Jordan R., Wong S.J. (2022). Association of plasma tumor tissue modified viral HPV DNA (TTMV) with tumor burden, treatment type, and outcome: A translational analysis from NRG-HN002. J. Clin. Oncol..

[34] Pfister D.G., Spencer S., Adelstein D., Adkins D., Anzai Y., Brizel D.M., Bruce J.Y., Busse P.M., Caudell J.J., Cmelak A.J. (2020). Head and neck cancers, version 2.2020, NCCN Clinical Practice Guidelines in Oncology. J. Natl. Compr. Cancer Netw..

[35] Cheung P.K.F., Chin R.Y., Eslick G.D. (2016). Detecting residual/recurrent head neck squamous cell carcinomas using PET or PET/CT: Systematic review and meta-analysis. Otolaryngol.–Head. Neck Surg..

[36] de Ridder M., Gouw Z.A.R., Navran A., Hamming-Vrieze O., Jasperse B., van den Brekel M.W.M., Zuur C.L., Hoekstra O.S., Comans E.F.I., van Velden F.H.P. (2019). FDG-PET/CT improves detection of residual disease and reduces the need for examination under anaesthesia in oropharyngeal cancer patients treated with (chemo-)radiation. Eur. Arch. Oto-Rhino-Laryngol..

[37] Ho A.S., Tsao G.J., Chen F.W., Shen T., Kaplan M.J., Colevas A.D., Fischbein N.J., Iagaru A., Laramore G.E., Quon A. (2013). Impact of positron emission tomography/computed tomography surveillance at 12 and 24 months for detecting head and neck cancer recurrence. Cancer.

[38] Li W., Chen J., Liang B., Li Z., Li J., Yuan X., Zhang M., Liu Y., Peng Y., Zhang F. (2021). Long-term monitoring of dynamic changes in plasma EBV DNA for improved prognosis prediction of nasopharyngeal carcinoma. Cancer Med..

[39] Lee A.W.M., Lee V.H.F., Ng W.T., Strojan P., Saba N.F., Rinaldo A., Genden E.M., Ferlito A., Kowalski L.P., Mendenhall W.M. (2021). A systematic review and recommendations on the use of plasma EBV DNA for nasopharyngeal carcinoma. Eur. J. Cancer.

[40] Colevas A.D. (2022). HPV DNA as a Biomarker in Oropharyngeal Cancer: A Step in the Right Direction. Clin. Cancer Res..

[41] Ekanayake Weeramange C., Liu Z., Hartel G., Li Y., Vasani S., Langton-Lockton J., Kenny L., Morris L., Frazer I., Tang K.D. (2021). Salivary high-risk human papillomavirus (HPV) DNA as a biomarker for HPV-driven head and neck cancers. J. Mol. Diagn. J. Mod. Dynam..

[42] Bettampadi D., Villa L.L., Ponce E.L., Salmeron J., Sirak B.A., Abrahamsen M., Rathwell J.A., Reich R.R., Giuliano A.R. (2020). Oral human papillomavirus prevalence and type distribution by country (Brazil, Mexico and the United States) and age among HPV infection in men study participants. Int. J. Cancer.

[43] Martin-Gomez L., Fulp W.J., Schell M.J., Sirak B., Abrahamsen M., Isaacs-Soriano K.A., Lorincz A., Wenig B., Chung C.H., Caudell J.J. (2019). Oral gargle-tumor biopsy human papillomavirus (HPV) agreement and associated factors among oropharyngeal squamous cell carcinoma (OPSCC) cases. Oral Oncol..

[44] Martin-Gomez L., Fulp W.J., Schell M.J., Sirak B., Abrahamsen M., Isaacs-Soriano K.A., Lorincz A., Wenig B., Chung C.H., Caudell J.J. (2022). Performance of oral HPV DNA, oral HPV mRNA and circulating tumor HPV DNA in the detection of HPV-related oropharyngeal cancer and cancer of unknown primary. Int. J. Cancer.

[45] Fakhry C., Blackford A.L., Neuner G., Xiao W., Jiang B., Agrawal A., Gillison M.L. (2019). Association of oral human papillomavirus DNA persistence with cancer progression after primary treatment for oral cavity and oropharyngeal squamous cell carcinoma. JAMA Oncol..

[46] Brakenhoff R.H., Wagner S., Klussmann J.P. (2017). Molecular patterns and biology of HPV-associated HNSCC. Recent Results Cancer Res..

[47] D’Souza G., Clemens G., Troy T., Castillo R.G., Struijk L., Waterboer T., Bender N., Pierorazio P.M., Haddad R.I., Zevallos J.P. (2019). Evaluating the Utility and Prevalence of HPV Biomarkers in Oral Rinses and Serology for HPV-Related Oropharyngeal Cancer. Cancer Prev. Res..

[48] Sano D., Oridate N. (2016). The molecular mechanism of human papillomavirus-induced carcinogenesis in head and neck squamous cell carcinoma. Int. J. Clin. Oncol..

[49] Solomon B., Young R.J., Rischin D. (2018). Head and neck squamous cell carcinoma: Genomics and emerging biomarkers for immunomodulatory cancer treatments. Semin. Cancer Biol..

[50] Lechner M., Fenton T.R. (2016). The genomics, epigenomics, and transcriptomics of HPV-associated oropharyngeal cancer–understanding the basis of a rapidly evolving disease. Adv. Genet..

[51] Gaździcka J., Gołąbek K., Strzelczyk J.K., Ostrowska Z. (2020). Epigenetic modifications in head and neck cancer. Biochem. Genet..

[52] Hill M., Tran N. (2021). miRNA interplay: Mechanisms and consequences in cancer. Dis. Model. Mech..

[53] Emmett S., Whiteman D.C., Panizza B.J., Antonsson A. (2018). An update on cellular MicroRNA expression in human papillomavirus-associated head and neck squamous cell carcinoma. Oncology.

[54] Kalfert D., Pesta M., Kulda V., Topolcan O., Ryska A., Celakovsky P., Laco J., Ludvikova M. (2015). MicroRNA profile in site-specific head and neck squamous cell cancer. Anticancer Res..

[55] Emmett S.E., Stark M.S., Pandeya N., Panizza B., Whiteman D.C., Antonsson A. (2021). MicroRNA expression is associated with human papillomavirus status and prognosis in mucosal head and neck squamous cell carcinomas. Oral Oncol..

[56] Nunvar J., Pagacova L., Vojtechova Z., Azevedo N.T.D., Smahelova J., Salakova M., Tachezy R., Betka J., Klozar J., Grega M. (2021). Lack of conserved miRNA deregulation in HPV-induced squamous cell carcinomas. Biomolecules.

[57] Guo D., Yang M., Li S., Zhu W., Chen M., Pan J., Long D., Liu Z., Zhang C. (2023). Expression and molecular regulation of non-coding RNAs in HPV-positive head and neck squamous cell carcinoma. Front. Oncol..

[58] Gougousis S., Mouchtaropoulou E., Besli I., Vrochidis P., Skoumpas I., Constantinidis I. (2020). HPV-related oropharyngeal cancer and biomarkers based on epigenetics and microbiome profile. Front. Cell Dev. Biol..

[59] Lajer C.B., Garnæs E., Friis-Hansen L., Norrild B., Therkildsen M.H., Glud M., Rossing M., Lajer H., Svane D., Skotte L. (2012). The role of miRNAs in human papilloma virus (HPV)-associated cancers: Bridging between HPV-related head and neck cancer and cervical cancer. Br. J. Cancer.

[60] Lajer C.B., Nielsen F.C., Friis-Hansen L., Norrild B., Borup R., Garnæs E., Rossing M., Specht L., Therkildsen M.H., Nauntofte B. (2011). Different miRNA signatures of oral and pharyngeal squamous cell carcinomas: A prospective translational study. Br. J. Cancer.

[61] Vojtechova Z., Sabol I., Salakova M., Smahelova J., Zavadil J., Turek L., Grega M., Klozar J., Prochazka B., Tachezy R. (2016). Comparison of the miRNA profiles in HPV-positive and HPV-negative tonsillar tumors and a model system of human keratinocyte clones. BMC Cancer.

[62] McCombie W.R., McPherson J.D., Mardis E.R. (2019). Next-generation sequencing technologies. Cold Spring Harb. Perspect. Med..

[63] Liu Z.H., Chen L.D., He Y.B., Xu B., Wang K.B., Sun G.X., Zhang Z.H. (2019). Levelsof expression levels and clinical significance of miR-503 and miR-375 in patients with esophageal squamous cell carcinoma. Eur. Rev. Med. Pharmacol. Sci..

[64] House R., Majumder M., Janakiraman H., Ogretmen B., Kato M., Erkul E., Longnecker R., Kellermayer R., Lee H., Sampson L. (2018). Smoking-induced control of miR-133a-3p alters the expression of EGFR and HuR in HPV-infected oropharyngeal cancer. PLoS ONE.

[65] Weiss B.G., Anczykowski M.Z., Ihler F., Bertlich M., Spiegel J.L., Haubner F., Przypadlo C.M., Heindl L.M., Hoffmann T.K., Sauter A. (2022). MicroRNA-182-5p and microRNA-205-5p as potential biomarkers for prognostic stratification of p16-positive oropharyngeal squamous cell carcinoma. Cancer Biomark..

[66] Bersani C., Mints M., Tertipis N., Haeggblom L., Näsman A., Romanitan M., Marklund L., Dalianis T., Ramqvist T., Munck-Wikland E. (2018). MicroRNA-155, -185 and -193b as biomarkers in human papillomavirus positive and negative tonsillar and base of tongue squamous cell carcinoma. Oral Oncol..

[67] Zhang C., Chen H., Deng Z., Long D., Xu L., Liu Z. (2020). DGCR8/miR-106 axis enhances radiosensitivity of head and neck squamous cell carcinomas by downregulating RUNX3. Front. Med..

[68] Casarotto M., Fanetti G., Guerrieri R., Palazzari E., Lupato V., Steffan A., Baggio V., Del Vecchio C., Giorgi C., Barzan L. (2020). Beyond MicroRNAs: Emerging role of other non-coding RNAs in HPV-driven cancers. Cancers.

[69] Castro-Oropeza R., Piña-Sánchez P. (2022). Epigenetic and transcriptomic regulation landscape in HPV+ cancers: Biological and clinical implications. Front. Genet..

[70] Luo X.J., Zheng M., Cao M.X., Zhang W.L., Huang M.C., Dai L., Wu S.Y., Liu M.Z., Liao X.B., Wang H.Y. (2020). Distinguishable prognostic miRNA signatures of head and neck squamous cell cancer with or without HPV infection. Front. Oncol..

[71] Salazar-Ruales C., Arguello J.V., López-Cortés A., Cabrera-Andrade A., García-Cárdenas J.M., Guevara-Ramírez P., Leone P.E., Paz-Y-Miño C., Ortiz M., Cabrera A. (2018). Salivary MicroRNAs for early detection of head and neck squamous cell carcinoma: A case-control study in the high altitude mestizo Ecuadorian population. BioMed Res. Int..

[72] Sannigrahi M.K., Sharma R., Singh V., Panda N.K., Rattan V., Khullar M. (2017). Role of host miRNA hsa-miR-139-3p in HPV-16-Induced carcinomas. Clin. Cancer Res..

[73] Orosz E., Gombos K., Petrevszky N., Csonka D., Haber I., Kaszas B., Gergely L., Csereklyei M., Bajzik G., Polgar C. (2020). Visualization of mucosal field in HPV positive and negative oropharyngeal squamous cell carcinomas: Combined genomic and radiology based 3D model. Sci. Rep..

[74] He H., Liu X., Liu Y., Zhang M., Lai Y., Hao Y., Wang Q., Shi D., Wang N., Luo X.G. (2019). Human Papillomavirus E6/E7 and Long Noncoding RNA TMPOP2 Mutually Upregulated Gene Expression in Cervical Cancer Cells. J. Virol..

[75] Shen L., Li N., Zhou Q., Li Z., Shen L. (2021). Development and validation of an autophagy-related LncRNA prognostic signature in head and neck squamous cell carcinoma. Front. Oncol..

[76] Chen L., Zhou Y., Sun Q., Zhou J., Pan H., Sui X. (2017). Regulation of autophagy by MiRNAs and their emerging roles in tumorigenesis and cancer treatment. Int. Rev. Cell Mol. Biol..

[77] Aranda-Rivera A.K., Cruz-Gregorio A., Briones-Herrera A., Pedraza-Chaverri J. (2021). Regulation of autophagy by high- and low-risk human papillomaviruses. Rev. Med. Virol..

[78] Vahabi M., Pulito C., Sacconi A., Donzelli S., D’Andrea M., Manciocco V., Pellini R., Pacini L., Sanguineti G., Strigari L. (2019). miR-96-5p targets PTEN expression affecting radio-chemosensitivity of HNSCC cells. J. Exp. Clin. Cancer Res..

[79] Long D., Xu L., Deng Z., Guo D., Zhang Y., Liu Z., Wang Y., Shi H., Zhang L., Liu J. (2021). HPV16 E6 enhances the radiosensitivity in HPV-positive human head and neck squamous cell carcinoma by regulating the miR-27a-3p/SMG1 axis. Infect. Agent. Cancer.

[80] Inoue H., Hirasaki M., Kogashiwa Y., Kuba K., Ebihara Y., Nakahira M., Saito K., Onitsuka T., Nakashima T. (2021). Predicting the radiosensitivity of HPV-negative oropharyngeal squamous cell carcinoma using miR-130b. Acta Otolaryngol..

[81] Fu E., Liu T., Yu S., Chen X., Song L., Lou H., Sun Y., Shi Q., Zhao Y., Li J. (2020). M2 macrophages reduce the radiosensitivity of head and neck cancer by releasing HB−EGF. Oncol. Rep..

[82] Feng Q., Zhang H., Yao D., Chen W.D., Wang Y.D. (2019). Emerging Role of Non-Coding RNAs in Esophageal Squamous Cell Carcinoma. Int. J. Mol. Sci..

[83] Dai D., Feng X.D., Zhu W.Q., Bao Y.N. (2019). LncRNA BLACAT1 regulates the viability, migration and invasion of oral squamous cell carcinoma cells by targeting miR-142-5p. Eur. Rev. Med. Pharmacol. Sci..

[84] Ma X., Zhang Q., Du J., Tang J., Tan B. (2021). Integrated analysis of ceRNA regulatory network associated with tumor stage in cervical cancer. Front. Genet..

[85] Kopczyńska M., Kolenda T., Guglas K., Sobocińska J., Teresiak A., Bliźniak R., Mackiewicz A., Lamperska K. (2020). PRINS lncRNA is a new biomarker candidate for HPV infection and prognosis of head and neck squamous cell carcinomas. Diagnostics.

[86] Chen X., Liu Y., Liu H., Wang Z.W., Zhu X. (2022). Unraveling diverse roles of noncoding RNAs in various human papillomavirus negative cancers. Pharmacol. Ther..

[87] Wang Z., Liu T., Li G., Cao Z. (2020). The exploration of new therapeutic targets for HPV-negative head and neck squamous cell cancer through the construction of a ceRNA network and immune microenvironment analysis. J. Cell Biochem..

[88] Haque S.U., Niu L., Kuhnell D., Hendershot J., Biesiada J., Niu W., Xu L., Sun W., Feng C., Peng S. (2018). Differential expression and prognostic value of long non-coding RNA in HPV-negative head and neck squamous cell carcinoma. Head. Neck.

[89] Yang Y., Feng L., Wang R., Ma H., He S., Fang J. (2022). Integrated analysis of lncRNA-associated ceRNA network in p16-positive and p16-negative head and neck squamous cell carcinoma. Medicine.

[90] Mainguené J., Vacher S., Kamal M., Hamza A., Masliah-Planchon J., Baulande S., Nicolas A., Couturier J., Pierron G., Le Tourneau C. (2022). Human papilloma virus integration sites and genomic signatures in head and neck squamous cell carcinoma. Mol. Oncol..

[91] Zhong Z., Hong M., Chen X., Xi Y., Xu Y., Kong D., Deng J., Li Y., Hu R., Sun C. (2020). Transcriptome analysis reveals the link between lncRNA-mRNA co-expression network and tumor immune microenvironment and overall survival in head and neck squamous cell carcinoma. BMC Med. Genom..

[92] Guo Y., Pan W.K., Wang Z.W., Su W.H., Xu K., Jia H., Li M.M., Liu H., Zhang B., Li Q. (2021). Identification of novel biomarkers for predicting prognosis and immunotherapy response in head and neck squamous cell carcinoma based on ceRNA network and immune infiltration analysis. BioMed Res. Int..

[93] Song L., Xie H., Tong F., Yan B., Zhang S., Fu E., Liu H., Lou H., Li X., Shi Q. (2019). Association of lnc-IL17RA-11 with increased radiation sensitivity and improved prognosis of HPV-positive HNSCC. J. Cell Biochem..

[94] Meng X., Lou Q.Y., Yang W.Y., Wang Y.R., Chen R., Wang L., Xu T., Zhang L. (2021). The role of non-coding RNAs in drug resistance of oral squamous cell carcinoma and therapeutic potential. Cancer Commun..

[95] Zhang H., Si J., Yue J., Ma S. (2021). The mechanisms and reversal strategies of tumor radioresistance in esophageal squamous cell carcinoma. J. Cancer Res. Clin. Oncol..

[96] Ma X., Sheng S., Wu J., Jiang Y., Gao X., Cen X., Zhang Y., Wang Z., Chen G., Hu Y. (2017). LncRNAs as an intermediate in HPV16 promoting myeloid-derived suppressor cell recruitment of head and neck squamous cell carcinoma. Oncotarget.

[97] Dias T.R., Santos J.M.O., Estêvão D., Costa N.R., Mestre V.F., Medeiros-Fonseca B., Marques R., Gil da Costa R.M., Lopes C., Oliveira P.A. (2022). Expression of LncRNAs in HPV-induced carcinogenesis and cancer cachexia: A study in K14-HPV16 mice. Anticancer Res..

[98] Ghafouri-Fard S., Hussen B.M., Shaterabadi D., Abak A., Shoorei H., Taheri M., Mokhtari M.J., Mohammadi-Yeganeh S., Savadi-Shiraz E., Hashemzadeh-Chaleshtori M. (2022). The interaction between human papilloma viruses related cancers and non-coding RNAs. Pathol. Res. Pract..

[99] Salinas-Montalvo A.M., Supramaniam A., McMillan N.A., Idris A. (2021). RNA-Based gene targeting therapies for human papillomavirus driven cancers. Cancer Lett..

[100] Huang Z.L., Chen R.P., Zhou X.T., Zhan H.L., Hu M.M., Liu B., Zhang Y.Y., Lei Z., Xie L., Chen M. (2017). Long non-coding RNA MEG3 induces cell apoptosis in esophageal cancer through endoplasmic reticulum stress. Oncol. Rep..

[101] Sannigrahi M.K., Sharma R., Panda N.K., Khullar M. (2018). Role of non-coding RNAs in head and neck squamous cell carcinoma: A narrative review. Oral. Dis..

[102] Chen Y., Luo T.Q., Xu S.S., Chen C.Y., Sun Y., Lin L., Zhang X., Xie J., Li C., Tang L. (2021). An immune-related seven-lncRNA signature for head and neck squamous cell carcinoma. Cancer Med..

[103] Guo Y., Yang P.T., Wang Z.W., Xu K., Kou W.H., Luo H. (2020). Identification of three autophagy-related long non-coding RNAs as a novel head and neck squamous cell carcinoma prognostic signature. Front. Oncol..

[104] Wu S., Huang X., Tie X., Cheng Y., Xue X., Fan M. (2021). Role and mechanism of action of circular RNA and laryngeal cancer. Pathol. Res. Pract..

[105] Qi X., Zhang D.H., Wu N., Xiao J.H., Wang X., Ma W. (2015). ceRNA in cancer: Possible functions and clinical implications. J. Med. Genet..

[106] Kristensen L.S., Jakobsen T., Hager H., Kjems J. (2022). The emerging roles of circRNAs in cancer and oncology. Nat. Rev. Clin. Oncol..

[107] Tornesello M.L., Faraonio R., Buonaguro L., Annunziata C., Starita N., Cerasuolo A., Pezzuto F., Tornesello A.L., Buonaguro F.M., Botti G. (2020). The role of microRNAs, long non-coding RNAs, and circular RNAs in cervical cancer. Front. Oncol..

[108] Jun W., Shaobo O., Zhang X., Zhao S., Chen M., Fan X., Cai Y., Lan L. (2021). Deregulation of hsa_circ_0001971/miR-186 and hsa_circ_0001874/miR-296 signaling pathways promotes the proliferation of oral squamous carcinoma cells by synergistically activating SHP2/PLK1 signals. Sci. Rep..

[109] Chen X., Yu J., Tian H., Shan Z., Liu W., Pan Z., Ren X., Li A., Wang X., Xie C. (2019). Circle RNA hsa_circRNA_100290 serves as a ceRNA for miR-378a to regulate oral squamous cell carcinoma cells growth via glucose transporter-1 (GLUT1) and glycolysis. J. Cell Physiol..

[110] Bonelli P., Borrelli A., Tuccillo F.M., Buonaguro F.M., Tornesello M.L. (2021). The role of circRNAs in human papillomavirus (HPV)-associated cancers. Cancers.

[111] Zhao S.Y., Wang J., Ouyang S.B., Huang Z.K., Liao L. (2018). Salivary circular RNAs Hsa_Circ_0001874 and Hsa_Circ_0001971 as novel biomarkers for the diagnosis of oral squamous cell carcinoma. Cell Physiol. Biochem..

[112] Zhao W., Cui Y., Liu L., Qi X., Liu J., Ma S., Zhou Q., Cao Q., Nie J., Li Y. (2020). Splicing factor derived circular RNA circUHRF1 accelerates oral squamous cell carcinoma tumorigenesis via feedback loop. Cell Death Differ..

[113] Cristóbal I., Caramés C., Rubio J., Sanz-Alvarez M., Luque M., Madoz-Gúrpide J., García-Foncillas J. (2020). Functional and clinical impact of CircRNAs in oral cancer. Cancers.

[114] Liu Y., Dou M., Song X., Dong Y., Liu S., Liu H., Tao J., Li W., Li H., Xie R. (2019). The emerging role of the piRNA/piwi complex in cancer. Mol. Cancer.

[115] Krishnan A.R., Qu Y., Li P.X., Zou A.E., Califano J.A., Wang-Rodriguez J., Gokhale P.C., Gachechiladze M.A., Ozturk K., Rubenstein M. (2018). Computational methods reveal novel functionalities of PIWI-interacting RNAs in human papillomavirus-induced head and neck squamous cell carcinoma. Oncotarget.

[116] Zou A.E., Zheng H., Saad M.A., Rahimy M., Ku J., Kuo S.Z., Honda T.K., Wang-Rodriguez J., Xuan Y., Korrapati A. (2016). The non-coding landscape of head and neck squamous cell carcinoma. Oncotarget.

[117] Firmino N., Martinez V.D., Rowbotham D.A., Enfield K.S.S., Bennewith K.L., Lam W.L. (2016). HPV status is associated with altered PIWI-interacting RNA expression pattern in head and neck cancer. Oral. Oncol..

[118] Xing L., Zhang X., Zhang X., Tong D. (2020). Expression scoring of a small-nucleolar-RNA signature identified by machine learning serves as a prognostic predictor for head and neck cancer. J. Cell Physiol..

[119] Paver E.C., Currie A.M., Gupta R., Dahlstrom J.E. (2020). Human Papilloma Virus Related Squamous Cell Carcinomas of the Head and Neck: Diagnosis, Clinical Implications and Detection of HPV. Pathology.

[120] Rahimi S. (2020). HPV-Related Squamous Cell Carcinoma of Oropharynx: A Review. J. Clin. Pathol..

[121] Lewis J.S. (2016). Sinonasal Squamous Cell Carcinoma: A Review with Emphasis on Emerging Histologic Subtypes and the Role of Human Papillomavirus. Head Neck Pathol..

[122] Švajdler M., Němcova J., Dubinský P., Metelkova A., Švajdler P., Straka Ľ., Saláková M., Kment M., Michálková R., Boudová L. (2021). Significance of Transcriptionally-Active High-Risk Human Papillomavirus in Sinonasal Squamous Cell Carcinoma: Case Series and a Meta-Analysis. Neoplasma.

[123] Prigge E., Arbyn M., von Knebel Doeberitz M., Reuschenbach M. (2017). Diagnostic Accuracy of P16ink4a Immunohistochemistry in Oropharyngeal Squamous Cell Carcinomas: A Systematic Review and Meta-Analysis. Int. J. Cancer.

[124] Menegaldo A., Schroeder L., Holzinger D., Tirelli G., Cin E.D., Tofanelli M., Giorgi Rossi P., Boscolo-Rizzo P., Da Mosto M.C., Del Mistro A. (2021). Detection of HPV16/18 E6 Oncoproteins in Head and Neck Squamous Cell Carcinoma Using a Protein Immunochromatographic Assay. Laryngoscope.

[125] Chernesky M., Jang D., Schweizer J., Arias M., Doerwald-Munoz L., Gupta M., Ely S., Borkowski R., Day A., Varley C. (2018). HPV E6 Oncoproteins and Nucleic Acids in Neck Lymph Node Fine Needle Aspirates and Oral Samples From Patients with Oropharyngeal Squamous Cell Carcinoma. Papillomavirus Res..

[126] Augustin J.G., Lepine C., Morini A., Brunet A., Veyer D., Brochard C., Lévêque N., Mirghani H., Pecking M., Temam S. (2020). HPV Detection in Head and Neck Squamous Cell Carcinomas: What Is the Issue?. Front. Oncol..

[127] Williams J., Kostiuk M., Biron V.L. (2022). Molecular Detection Methods in HPV-Related Cancers. Front. Oncol..

[128] Jeannot E., Latouche A., Bonneau C., Calméjane M.-A., Beaufort C.M., Ruigrok-Ritstier K., Bernard-Tessier A., Bossard C., Malaquin N., Rouzier R. (2021). Circulating HPV DNA as a Marker for Early Detection of Relapse in Patients with Cervical Cancer. Clin. Cancer Res..

[129] Krasniqi E., Barba M., Venuti A., Pizzuti L., Cappuzzo F., Landi L., Carpano S., Marchetti P., Villa A., Vizza E. (2021). Circulating HPV DNA in the Management of Oropharyngeal and Cervical Cancers: Current Knowledge and Future Perspectives. J. Clin. Med..

[130] Taylor S.C., Laperriere G., Germain H. (2017). Droplet Digital PCR Versus qPCR for Gene Expression Analysis with Low Abundant Targets: From Variable Nonsense to Publication Quality Data. Sci. Rep..

[131] Li H., Bai R., Zhao Z., Tao L., Ma M., Ji Z., Li X., Zhang H., Sun J., Wang J. (2018). Application of Droplet Digital PCR to Detect the Pathogens of Infectious Diseases. Biosci. Rep..

[132] Malin K., Louise B.M., Gisela H., Mats K.G., Gabriella L.-L. (2021). Optimization of Droplet Digital PCR Assays for the Type-Specific Detection and Quantification of Five HPV Genotypes, Including Additional Data on Viral Loads of Nine Different HPV Genotypes in Cervical Carcinomas. J. Virol. Methods.

[133] Hayden J.P., Wiggins A., Sullivan T., Kalantzakos T., Hooper K., Moinzadeh A., Rieger-Christ K. (2024). Use of Droplet Digital Polymerase Chain Reaction to Identify Biomarkers for Differentiation of Benign and Malignant Renal Masses. Cancers.

[134] Larsson G.L., Helenius G. (2017). Digital Droplet PCR (ddPCR) for the Detection and Quantification of HPV 16, 18, 33 and 45—A Short Report. Cell. Oncol..

[135] Rotondo J.C., Oton-Gonzalez L., Mazziotta C., Lanzillotti C., Iaquinta M.R., Tognon M., Martini F., Contini C. (2020). Simultaneous Detection and Viral DNA Load Quantification of Different Human Papillomavirus Types in Clinical Specimens by the High Analytical Droplet Digital PCR Method. Front. Microbiol..

[136] Amin M.B., Greene F.L., Edge S.B., Compton C.C., Gershenwald J.E., Brookland R.K., Meyer L., Gress D.M., Byrd D.R., Winchester D.P. (2017). The Eighth Edition AJCC Cancer Staging Manual: Continuing to build a bridge from a population-based to a more “personalized” approach to cancer staging. CA Cancer J. Clin..

[137] Hsieh J.C.H., Wang H.M., Wu M.H., Chang K.P., Chang P.H., Liao C.T., Chen I.H., Yen T.C., Lin C.Y., Lin J.T. (2019). Review of emerging biomarkers in head and neck squamous cell carcinoma in the era of immunotherapy and targeted therapy. Head Neck.

[138] Barber B.R., Biron V.L., Klimowicz A.C., Puttagunta L., Côté D.W.J., Seikaly H. (2013). Molecular predictors of locoregional and distant metastases in oropharyngeal squamous cell carcinoma. J. Otolaryngol. Head Neck Surg..

[139] Mehrad M., Zhao H., Gao G., Wang X., Lewis J.S. (2014). Transcriptionally-active human papillomavirus is consistently retained in the distant metastases of primary oropharyngeal carcinomas. Head Neck Pathol..

[140] Lewis J.S. (2020). Human papillomavirus testing in head and neck squamous cell carcinoma in 2020: Where are we now and where are we going?. Head Neck Pathol..

[141] Galati L., Chiocca S., Duca D., Tagliabue M., Simoens C., Gheit T., Arbyn M., Tommasino M. (2022). HPV and head and neck cancers: Towards early diagnosis and prevention. Tumor Virus Res..

[142] Mes S.W., Heideman D.A.M., Bloemena E., Brink A., Bogaarts M., Leemans C.R., Brakenhoff R.H. (2020). Development and validation of a novel and rapid molecular detection method for high-risk human papillomavirus in formalin-fixed, paraffin-embedded tumor tissue. J. Mol. Diagn. J. Mod. Dynam..

[143] von Knebel Doeberitz M. (2016). The causal role of human papillomavirus infections in non-anogenital cancers. It’s time to ask for the functional evidence. Int. J. Cancer.

[144] Chera B.S., Amdur R.J., Green R., Shen C., Gupta G., Tan X., Knowles M., Fried D., Hayes N., Weiss J. (2019). Phase II trial of de-intensified chemoradiotherapy for human papillomavirus-associated oropharyngeal squamous cell carcinoma. J. Clin. Oncol. Off. J. Am. Soc. Clin. Oncol..

[145] Deng Z., Hasegawa M., Aoki K., Matayoshi S., Kiyuna A., Yamashita Y., Uehara T., Agena S., Maeda H., Suzuki M. (2014). A comprehensive evaluation of human papillomavirus positive status and p16INK4a overexpression as a prognostic biomarker in head and neck squamous cell carcinoma. Int. J. Oncol..

[146] Sabatini M.E., Chiocca S. (2020). Human papillomavirus as a driver of head and neck cancers. Br. J. Cancer.

[147] Simoens C., Gorbaslieva I., Gheit T., Tommasino M., Vorsters A., Lefevre K., Dorny P., Vanden Broeck D., Weyn C., Praet M. (2021). HPV DNA genotyping, HPV E6*I mRNA detection, and p16(INK4a)/Ki-67 staining in Belgian head and neck cancer patient specimens, collected within the HPV-AHEAD study. Cancer Epidemiol..

[148] Shinn J.R., Davis S.J., Lang-Kuhs K.A., Harris J.P., Chen M.M., Walter L.C., Habboushe J., Thakar S.D., Hara W., El-Sayed I.H. (2021). Oropharyngeal squamous cell carcinoma with discordant p16 and HPV mRNA results: Incidence and characterization in a large, contemporary United States cohort. Am. J. Surg. Pathol..

[149] Gheit T., Anantharaman D., Holzinger D., Alemany L., Tous S., Lucas E., Prabhu P.R., Pawlita M., Ridder R., Rehm S. (2017). Role of mucosal high-risk human papillomavirus types in head and neck cancers in central India. Int. J. Cancer.

[150] Ghouneimy A., Ali Z., Aman R., Jiang W., Aouida M., Mahfouz M. (2024). CRISPR-Based Multiplex Detection of Human Papillomaviruses for One-Pot Point-of-Care Diagnostics. ACS Synth. Biol..

[151] Cong L., Ran F.A., Cox D., Lin S., Barretto R., Habib N., Hsu P.D., Wu X., Jiang W., Marraffini L.A. (2013). Multiplex genome engineering using CRISPR/Cas systems. Science.

[152] Choudhary M.L., Anand S.P., Tikhe S.A., Walimbe A.M., Potdar V.A., Chadha M.S., Mishra A.C. (2016). Comparison of the conventional multiplex RT-PCR, real time RT-PCR and Luminex xTAG^®^ RVP fast assay for the detection of respiratory viruses. J. Med. Virol..

[153] Chen J.S., Ma E., Harrington L.B., Da Costa M., Tian X., Palefsky J.M., Doudna J.A. (2018). CRISPR-Cas12a target binding unleashes indiscriminate single-stranded Dnase activity. Science.

[154] Li L., Li S., Wu N., Wu J., Wang G., Zhao G., Wang J. (2019). HOLMESv2: A CRISPR-Cas12b- Assisted Platform for Nucleic Acid Detection and DNA Methylation Quantitation. ACS Synth. Biol..

[155] Gootenberg J.S., Abudayyeh O.O., Kellner M.J., Joung J., Collins J.J., Zhang F. (2018). Multiplexed and portable nucleic acid detection platform with Cas13, Cas12a, and Csm6. Science.

[156] Tian T., Qiu Z., Jiang Y., Zhu D., Zhou X. (2022). Exploiting the orthogonal CRISPR-Cas12a/Cas13a trans-cleavage for dual-gene virus detection using a handheld device. Biosens. Bioelectron..

[157] Abudayyeh O.O., Gootenberg J.S., Konermann S., Joung J., Slaymaker I.M., Cox D.B., Shmakov S., Makarova K.S., Semenova E., Minakhin L. (2016). C2c2 is a single-component programmable RNA-guided RNA-targeting CRISPR effector. Science.

[158] Dmytrenko O., Neumann G.C., Hallmark T., Keiser D.J., Crowley V.M., Vialetto E., Mougiakos I., Wandera K.G., Domgaard H., Weber J. (2023). Cas12a2 elicits abortive infection through RNA-triggered destruction of dsDNA. Nature.

[159] Mustafa M.I., Makhawi A.M. (2021). SHERLOCK and DETECTR: CRISPR-Cas Systems as Potential Rapid Diagnostic Tools for Emerging Infectious Diseases. J. Clin. Microbiol..

[160] Feng W., Newbigging A.M., Tao J., Cao Y., Peng H., Le C., Wu J., Pang B., Li J., Tyrrell D.L. (2021). CRISPR technology incorporating amplification strategies: Molecular assays for nucleic acids, proteins, and small molecules. Chem. Sci..

[161] Mabey D., Peeling R.W., Ustianowski A., Perkins M.D. (2004). Diagnostics for the developing world. Nat. Rev. Microbiol..

[162] Gong J., Zhang G., Wang W., Liang L., Li Q., Liu M., Xue L., Tang G. (2021). A simple and rapid diagnostic method for 13 types of high-risk human papillomavirus (HR-HPV) detection using CRISPR-Cas12a technology. Sci. Rep..

[163] Subica A.M. (2023). CRISPR in Public Health: The Health Equity Implications and Role of Community in Gene-Editing Research and Applications. Am. J. Public. Health.

[164] Kim N., Kim H.K., Lee S., Seo J.H., Choi J.W., Park J., Min S., Yoon S., Cho S.R., Kim H.H. (2020). Prediction of the Sequence-specific Cleavage Activity of Cas9 Variants. Nat. Biotechnol..

[165] Zhang G., Dai Z., Dai X. (2020). C-RNNCrispr: Prediction of CRISPR/Cas9 sgRNA activity using convolutional and recurrent neural networks. Comput. Struct. Biotechnol. J..

[166] Goodwin S., McPherson J.D., McCombie W.R. (2016). Coming of age: Ten years of next-generation sequencing technologies. Nat. Rev. Genet..

[167] Satam H., Joshi K., Mangrolia U., Waghoo S., Zaidi G., Rawool S., Thakare R.P., Banday S., Mishra A.K., Das G. (2023). Next-Generation Sequencing Technology: Current Trends and Advancements. Biology.

[168] Costain G., Cohn R.D., Scherer S.W., Marshall C.R. (2021). Genome sequencing as a diagnostic test. Can. Med. Assoc. J..

[169] Andersen K., Holm K., Tranberg M., Pedersen C.L., Bønløkke S., Steiniche T., Andersen B., Stougaard M. (2022). Targeted Next Generation Sequencing for Human Papillomavirus Genotyping in Cervical Liquid-Based Cytology Samples. Cancers.

[170] Chen J.-W., Shrestha L., Green G., Leier A., Marquez-Lago T.T. (2023). The hitchhikers’ guide to RNA sequencing and functional analysis. Brief. Bioinform..

[171] Stark R., Grzelak M., Hadfield J. (2019). RNA sequencing: The teenage years. Nat. Rev. Genet..

[172] Van den Bossche V., Zaryouh H., Vara-Messler M., Vignau J., Machiels J.P., Wouters A., Schmitz S., Corbet C. (2022). Microenvironment-driven intratumoral heterogeneity in head and neck cancers: Clinical challenges and opportunities for precision medicine. Drug Resist. Updat..

[173] Cho Y.U. (2024). The role of next-generation sequencing in hematologic malignancies. Blood Res..

[174] Singhal J., Verma S., Kumar S., Mehrotra D. (2021). Recent Advances in Nano-Bio-Sensing Fabrication Technology for the Detection of Oral Cancer. Mol. Biotechnol..

[175] Bartosik M., Moranova L., Izadi N., Strmiskova J., Sebuyoya R., Holcakova J., Hrstka R. (2024). Advanced technologies towards improved HPV diagnostics. J. Med. Virol..

[176] Safari M., Moghaddam A., Salehi Moghaddam A., Absalan M., Kruppke B., Ruckdäschel H., Khonakdar H.A. (2023). Carbon-based biosensors from graphene family to carbon dots: A viewpoint in cancer detection. Talanta.

[177] Laraib U., Sargazi S., Rahdar A., Khatami M., Pandey S. (2022). Nanotechnology-based approaches for effective detection of tumor markers: A comprehensive state-of-the-art review. Int. J. Biol. Macromol..

[178] Cheng C.S., Ou B.R., Lung F.D. (2022). Developing a Biosensor-Based Immunoassay to Detect HPV E6 Oncoprotein in the Saliva Rinse Fluid of Oral Cancer Patients. J. Pers. Med..

[179] Shigeishi H. (2023). Association between human papillomavirus and oral cancer: A literature review. Int. J. Clin. Oncol..

[180] Martínez-Barajas M.G., Jave-Suárez L.F., Ramírez-López I.G., García-Chagollán M., Zepeda-Nuño J.S., Ramírez-de-Arellano A., Ortiz-Lazareno P.C., Villegas-Pineda J.C., Pereira-Suárez A.L. (2023). HPV-Negative and HPV-Positive Oral Cancer Cells Stimulate the Polarization of Neutrophils towards Different Functional Phenotypes In Vitro. Cancers.

[181] Lyu X., Zhang M., Li G., Jiang Y., Qiao Q. (2019). PD-1 and PD-L1 Expression Predicts Radiosensitivity and Clinical Outcomes in Head and Neck Cancer and is Associated with HPV Infection. J. Cancer.

[182] Chen H., Zheng X., Li L., Huang L., Huang W., Ma Y. (2023). Peptide-Based Therapeutic HPV Cancer Vaccine Synthesized via Bacterial Outer Membrane Vesicles. Int. J. Nanomed..

[183] Mock A., Plath M., Moratin J., Tapken M.J., Jäger D., Krauss J., Fröhling S., Hess J., Zaoui K. (2021). EGFR and PI3K Pathway Activities Might Guide Drug Repurposing in HPV-Negative Head and Neck Cancers. Front. Oncol..

[184] Marquard F.E., Jücker M. (2020). PI3K/AKT/mTOR signaling as a molecular target in head and neck cancer. Biochem. Pharmacol..

[185] Ruffin A.T., Li H., Vujanovic L., Zandberg D.P., Ferris R.L., Bruno T.C. (2023). Improving head and neck cancer therapies by immunomodulation of the tumor microenvironment. Nat. Rev. Cancer..

